# Autophagy modulates growth and development in the moss *Physcomitrium patens*

**DOI:** 10.3389/fpls.2022.1052358

**Published:** 2022-12-19

**Authors:** Georgina Pettinari, Juan Finello, Macarena Plaza Rojas, Franco Liberatore, Germán Robert, Santiago Otaiza-González, Pilar Velez, Martin Theumer, Patricia Agudelo-Romero, Alejandro Enet, Claudio González, Ramiro Lascano, Laura Saavedra

**Affiliations:** ^1^ Unidad Ejecutora de Doble Dependencia INTA-CONICET (UDEA), Córdoba, Argentina; ^2^ Cátedra de Fisiología Vegetal, Facultad de Ciencias Exactas, Físicas y Naturales, Universidad Nacional de Córdoba, Córdoba, Argentina; ^3^ Centro de Investigaciones en Bioquímica Clínica e Inmunología (CIBICI), Universidad Nacional de Córdoba-CONICET, Córdoba, Argentina; ^4^ Telethon Kids Institute, Perth Children’s Hospital, Nedlands, Australia

**Keywords:** apical growth, nutrient starvation, bryophytes, autophagy, senescence, 2D and 3D growth and development, ATG8

## Abstract

*Physcomitrium patens* apical growing protonemal cells have the singularity that they continue to undergo cell divisions as the plant develops. This feature provides a valuable tool to study autophagy in the context of a multicellular apical growing tissue coupled to development. Herein, we showed that the core autophagy machinery is present in the moss *P. patens*, and characterized the 2D and 3D growth and development of *atg5* and *atg7* loss-of-function mutants under optimal and nutrient-deprived conditions. Our results showed that 2D growth of the different morphological and functional protonemata apical growing cells, chloronema and caulonema, is differentially modulated by this process. These differences depend on the protonema cell type and position along the protonemal filament, and growth condition. As a global plant response, the absence of autophagy favors the spread of the colony through protonemata growth at the expense of a reduction of the 3D growth, such as the buds and gametophore development, and thus the adult gametophytic and reproductive phases. Altogether this study provides valuable information suggesting that autophagy has roles during apical growth with differential responses within the cell types of the same tissue and contributes to life cycle progression and thus the growth and development of the 2D and 3D tissues of *P. patens*.

## 1 Introduction

Macroautophagy (hereafter autophagy) is an essential catabolic pathway for eukaryotic cell homeodynamics that mediates cellular recycling during growth, development and stress conditions (Marshall and Vierstra, 2018), involving the coordinated interaction among more than 30 highly conserved autophagy-related (*ATG*) proteins and components of the secretory system (Farhan et al., 2017; Soto-Burgos et al., 2018; Nakatogawa, 2020; Wang et al., 2020). During this process, a wide range of intracellular material is engulfed into double-membrane structures termed autophagosomes, and transported this autophagic cargo to vacuoles in plant and yeast cells (or lysosomes in mammalian cells), to be degraded and recycled for different cellular purposes or remobilized to other parts of the organism (Marshall and Vierstra, 2018).

Most knowledge in the field of plant autophagy arises from studies in Angiosperms, mainly in the model *Arabidopsis thaliana*, or crops such as *Zea mays*, *Oryza sativa*, and *Glycine max* (Tang and Bassham, 2018), pointing out that autophagy is virtually involved in all aspects of plant physiology (Doelling et al., 2002; Hanaoka et al., 2002; Kwon et al., 2010; Guiboileau et al., 2012; Avin-Wittenberg et al., 2015; Li et al., 2015; Estrada-Navarrete et al., 2016; Di Berardino et al., 2018; Sera et al., 2019; Li et al., 2020). Land plants (embryophytes) are a monophyletic lineage that evolved from within freshwater streptophyte algae c. 470–515 mya (Puttick et al., 2018). The recent availability of sequenced genomes from several lineages of streptophyte algae and bryophyte species has allowed comprehensive comparative evolutionary analyses moving forward in our understanding of plant evolution (Rensing, 2017; Fürst-Jansen et al., 2020). Studies on autophagy in the algae *Chlamydomonas reinhardtii* (Pérez-Pérez et al., 2017; Kajikawa and Fukuzawa, 2020), the liverwort *Marchantia polymorpha* (Norizuki et al., 2019), and the moss *Physcomitrium (Physcomitrella) patens* (Mukae et al., 2015; Sanchez-Vera et al., 2017; Chen et al., 2020; Kanne et al., 2021) have started to emerge, providing progress on evolutionary aspects of plant autophagy as well as species-specific roles of this process.

*P. patens* has been well-established as a model system (Rensing et al., 2020). It is also a model for polar growth studies because similar to unicellular root hairs and pollen tubes, rhizoids and protonemata exhibit apical growth (Vidali and Bezanilla, 2012; Rounds and Bezanilla, 2013), but in contrast to them, have the singularity that continues to undergo cell divisions as the plant develops, allowing to study this process in the context of a multicellular tissue (Rounds and Bezanilla, 2013). Protonemal cells live longer than pollen tubes and root hairs and integrate considerably more environmental signals during their growth and development (Bascom et al., 2018). Protonemata are composed of two distinct types of cells: chloronemata and caulonemata. Whereas chloronemata are rich in chloroplasts, divide every 24 hours, and have a photosynthetic role, caulonemata contain fewer and less developed chloroplasts, divide every 8 hours, and have a role in substrate colonization and nutrient acquisition (Menand et al., 2007a). Caulonema differentiates from a chloronemal tip cell, and this transition is regulated by auxin levels and energy availability (Thelander et al., 2005; Viaene et al., 2014; Thelander et al., 2018). Thus, caulonemata growth is favored by high light conditions or an external carbon source such as glucose (Thelander et al., 2005), but the development of this cell type is also triggered by nutrient deficiencies such as phosphate starvation (Wang et al., 2008) or growth with nitrate as a single nitrogen source, probably due to a carbon/nitrogen unbalance.

*P. patens atg5* and *atg7* loss-of-function mutants revealed that autophagy is needed for fertility, spermatid cytoplasmic clearance, and egg cavity mucilage formation (Sanchez-Vera et al., 2017). On the other hand, *PpATG8* overexpression enhanced the ability to reprogram somatic gametophore cells into chloronema stem cells when subjected to severe wounding (Kanne et al., 2021), whereas the absence of autophagy negatively affects this process (Rodriguez et al., 2020). Moreover, *atg3* loss-of-function lines showed premature gametophore senescence under non-stress conditions, and accumulated plastoglobuli suggesting that autophagy degradation of damaged chloroplasts in senescent gametophore cells is impaired in this mutant, (Chen et al., 2020). Using *atg5* loss-of-function lines Mukae et al., (Mukae et al., 2015) reported an early senescent phenotype under carbon and nitrogen starvation conditions, which was partly explained by amino acid imbalance because of the lack of cytoplasmic degradation by autophagy in *P. patens*. Despite these recent advances, there is a lack of studies considering temporal and spatial regulation of autophagy in *P. patens* apical growing cells. Thus, two unanswered questions are: (1) if the chloro- and caulonemata cells respond differentially to canonical autophagy-inducing conditions, and (2) how the absence of autophagy could affect the 2D growth and development of the juvenile protonemata and its transition to the 3D and gametophytic phase.

In this study, we described the ATG system in *P. patens* and explored the role of autophagy in the modulation of 2D and 3D growth and development under optimal, and carbon (C) and nitrogen (N) starvation conditions. Phenotypic characterization of *atg5*, and *atg7* loss-of-function lines combined with hormone analysis, and *PpATG8a-f* gene expression analysis under C and N starvation were performed. Likewise, induction of autophagy in protonemata was assayed by autophagic flux assays and visualization of autophagic vesicles using a *PpATG8e::GFP-PpATGe* reporter line. Our results showed that protonemata apical growth is differentially modulated by autophagy. These differences depend on the protonema cell type but also the position along the protonemal filament (apical vs. subapical) and growth condition. As a global plant response, the absence of autophagy prioritizes the spread of the colony through the 2D protonemata growth at the expense of a reduction in cell density and the development, given smaller gametophores with shorter rhizoids and early senescence, thus affecting the 3D development of adult gametophytic and reproductive life cycle progression. Altogether this study provides valuable information about autophagy roles during apical growth highlighting a degree of cell-type specificity in the autophagy response within the same tissue, which in turn affects the progression of the 2D and 3D tissues of *P. patens*.

## 2 Results

### 2.1 Differential development of *P. patens* in response to carbon and nitrogen starvation

**Figure 1:**
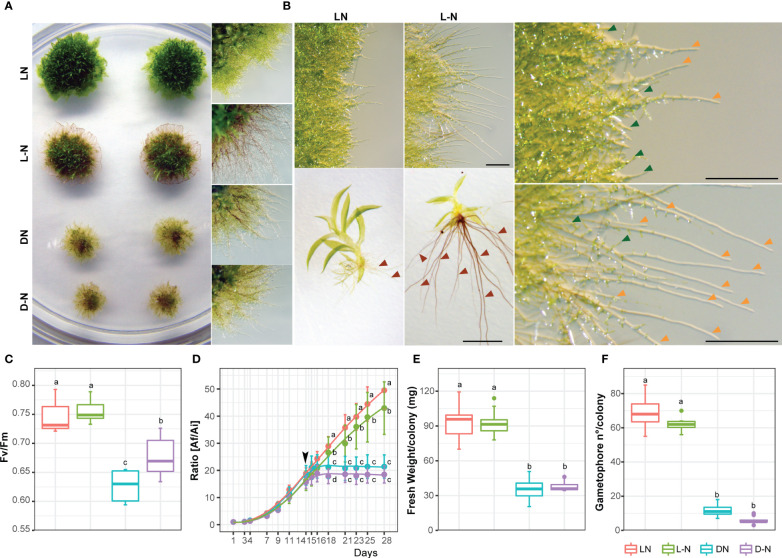
Effect of carbon and nitrogen deficiencies during a 14 day-period in colonies of *P. patens Grandsen*. **(A)** Representative images of *P. patens* colonies grown for 14 days in BCDAT (control media) and then subjected for 14 additional days to optimal growth conditions of light and nitrogen (LN), nitrogen deficiency (L-N), darkness (DN), or darkness and nitrogen deficiency (D-N). Colony view (left) and protonemata amplification view (right). **(B)** Representative images of protonemata after 5 days (upper panel and amplification in the right panel) and gametophores after 12 days (lower panel) of treatment to L-N or control LN. Green, orange, and red arrows indicate chloronemata, caulonemata, and rhizoids, respectively. Scale bar, 1 mm. **(C)** Box plot of PSII quantum yield (Fv/Fm). Letters indicate significant differences between treatments (*n*=6, one-way ANOVA and Tukey’s HSD test; *P* < 0.05). **(D)** Quantification of plant growth during the 28-day-old experiment was calculated as the ratio between the colony area at each time point (Af) and the colony initial area (Ai). The arrow indicates the time of transfer to deficiency media on day 14. Letters indicate significant differences between treatments for each time-point (*n*= 12, One-way ANOVA and Tukey’s HSD test; *P*< 0.05). **(E)** Quantification of colony fresh weight (mg). Letters indicate significant differences between treatments (*n*= 12, One-way ANOVA and Tukey’s HSD test; *P*< 0.001). **(F)** Quantification of gametophore number per colony, at the end of the treatment period, *n≥*6. Values represent the mean ± s.d. of biological replicates. Letters indicate significant differences between groups (one-way ANOVA and Tukey’s HSD test; *P* < 0.001).

To study responses to nutrient deficiencies, known to trigger autophagy and senescence in *P. patens*, colonies of wild-type *P. patens* were grown for 14 days under optimal growth conditions (BCDAT media, with nitrate and ammonium as nitrogen sources) and then kept under optimal growth and light conditions (LD photoperiod) either with or without nitrogen supply (nitrogen starvation), or transferred to darkness (carbon starvation), or darkness without nitrogen (carbon and nitrogen starvation) for additional 14 days. As shown in **Figure 1**, *P. patens* responded differentially to the mentioned treatments. Darkness with nitrogen (DN) or without nitrogen (D-N) induced senescence and cessation of growth and development, observed by the yellowed colony phenotype (**Figure 1A**), reduced values of maximum quantum efficiency PSII (Fv/Fm) (**Figure 1C**), reduced growth and colony fresh weight (**Figures 1D, E**), and a low number of developed gametophores (**Figure 1F**) in comparison to optimal growth conditions (LN). However, after 14 days in a nitrogen-deficient medium (L-N) no senescence symptoms (**Figure 1B**) or cessation of growth were observed, but rather a change in *P. patens* developmental pattern (**Figures 1B-F**). Five days after transferring to L-N conditions, more caulonemata were developed (**Figure 1B** upper panel), and on day 12 the gametophores exhibited longer and darker rhizoids with a reduced number of phyllids (**Figure 1B** lower panel), in comparison to the LN condition. These results indicate that darkness leads to rapid senescence, whereas under nitrogen deficiency other physiological and developmental strategies, known as phenotypic plasticity, are elicited in *P. patens* allowing growth and survival for longer periods.

### 2.2 The *atg5* and *atg7* mutants exhibit impaired growth and altered development under optimal growth conditions and hypersensibility to nutrient-deficient conditions

**Figure 2:**
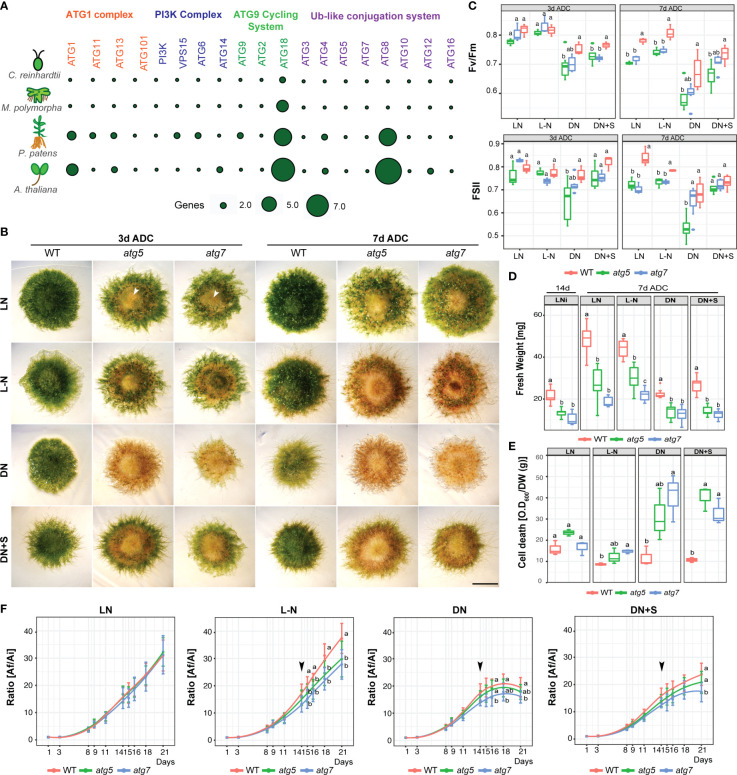
*P. patens atg5* and *atg7* mutants are hypersensitive to carbon and nitrogen starvation. **(A)** Comparison of the core autophagy genes (*ATG*) of *C. reinhardtii*, *M. polymorpha*, *P. patens*, and *A*. *thaliana.* **(B)** Representative images of *P. patens* colonies grown for 14 days in BCDAT (control media) and then subjected for 3 to 7 days to optimal growth conditions of light and nitrogen (LN), or nutrient deficient conditions (After Deficient Conditions, ADC) namely, nitrogen deficiency (L-N), darkness (DN), or darkness supplemented with 2% sucrose (DN+S). Scale bar, 5 mm. The white arrow indicates senescence in the center of the colony. **(C)** Photosynthetic parameters Fv/Fm and ΦPSII of wild type, *atg5*, and *atg7* mutants at 3 days ADC or 7 days. Letters indicate significant differences between genotypes within the same treatment (*n*= 9, One-way ANOVA and Tukey’s HSD test; *P*< 0.05). **(D)** Quantification of colony fresh weight (mg) after 14d in optimal growth conditions (LN) and at the end of the experiment, at day 21. Letters indicate significant differences between genotypes within the same treatment (*n*= 9, One-way ANOVA and Tukey’s HSD test; *P*< 0.05). **(E)** Quantification of colony cell death with Evans Blue 7 days ADC. Letters indicate significant differences between genotypes within the same treatment (*n*= 3, One-way ANOVA and Tukey’s HSD test; *P*< 0.05). **(F)** Quantification of plant growth during the 21-day-old experiment was calculated as the ratio between the colony area at each time point (Af) and the initial colony area (Ai). Letters indicate significant differences between genotypes for each time-point (*n*= 9, One-way ANOVA and Tukey’s HSD test; *P*< 0.05). The black arrow indicates the day when the plants were transferred to deficiency media. Values represent the mean ± s.d. of the biological replicates.

In order to study the functional role of specific autophagic genes (*ATG*) under nutrient stress, a search for *ATG* orthologs genes was performed in the *P. patens* genome using *A. thaliana ATG* genes as a query. All core autophagy genes are present in *P. patens* (**Table S1**), and specific gene families such as the *PpATG8* (**Figure S1**) and *PpATG18* contain several members (6 genes each), different from what is observed in *M. polymorpha* which harbors 2 *ATG8s*, 4 *ATG18s* and one gene for the other core autophagy machinery components (**Figure 2A**; **Table S2**), (Norizuki et al., 2019).

It is well known that ATG5 and ATG7 are indispensable for autophagic vesicle formation, being involved in the lipidation of ATG8 with phosphatidylethanolamine, and thus knocking out these genes results in the inhibition of autophagy in several organisms (Chung et al., 2010). To corroborate this in *P. patens a PpATG8e::GFP-PpATG8e* reporter line that expresses *GFP-PpATG8e* (**Table S1**) under the control of its endogenous promoter (Sanchez-Vera et al., 2017) was used to generate a null *atg5* mutant and confirmed that the autophagic flux was indeed inhibited under both optimal growth conditions and carbon starvation (**Figure S2**). Next, *atg5* and *atg7* null mutants were generated in *P. patens* by homologous recombination using targeted gene disruption in a wild-type background (**Figure S3**, **Table S3**).

**Figure 3:**
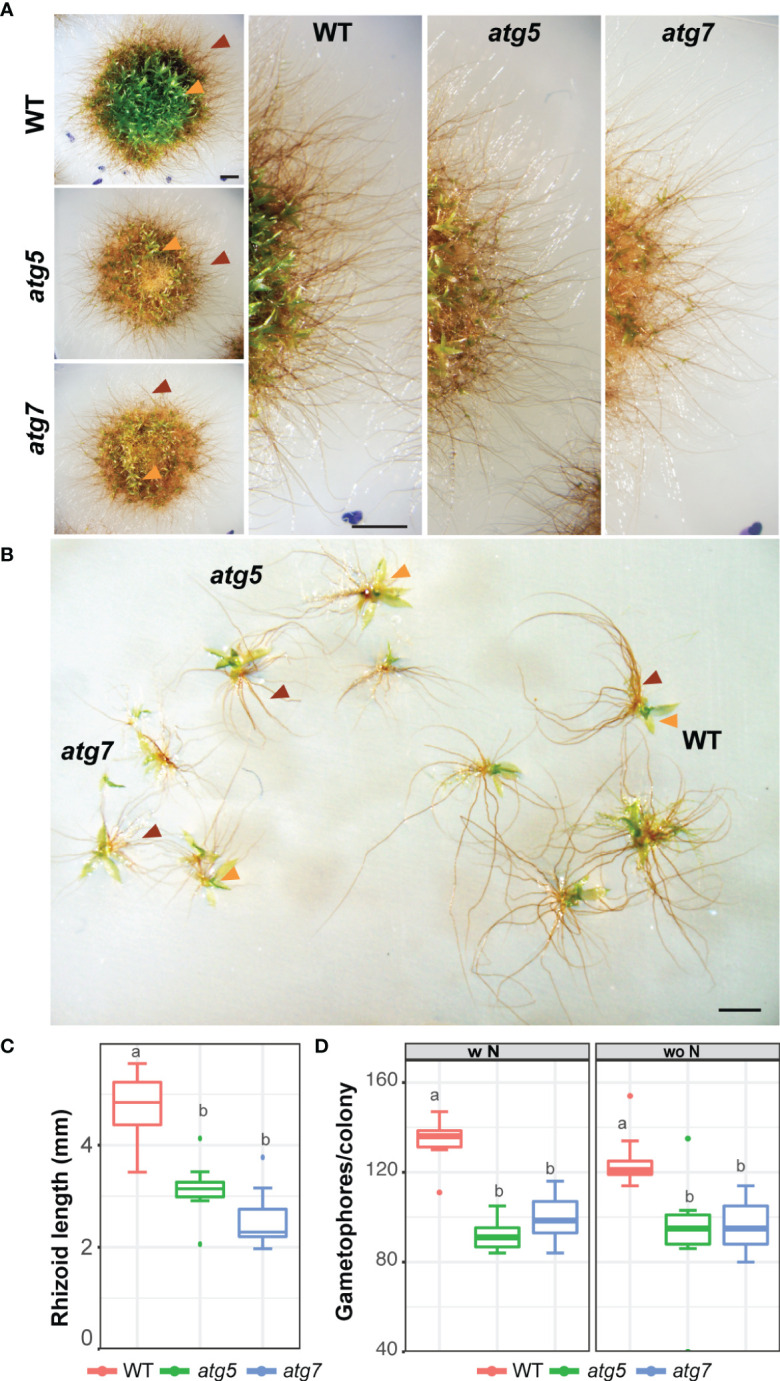
Morphological changes of *atg* mutants under nitrogen-deficient conditions. **(A)** Representative images of *P. patens* colonies grown for 14 days in BCDAT (control media) and then subjected for 7 additional days to nitrogen deficiency. Colony view (left), colony amplification view showing growing gametophores (right). **(B)** Representative gametophores from wild-type, *atg5*, and *atg7* knockout lines. **(C)** Quantification of gametophore rhizoid length (mm) at the end of the experiment (day 22). Letters indicate significant differences between genotypes (*n*= 8-10, One-way ANOVA and Tukey’s HSD test; P < 0.001). **(D)** Quantification of gametophore number at the end of the experiment (day 22). Letters indicate significant differences between genotypes and treatment (*n*= 9, Two-way ANOVA and Tukey’s HSD test; P < 0.01). Orange and red arrows indicate leafy gametophores and rhizoids, respectively.

The phenotype of *atg* lines was evaluated under carbon and nitrogen starvation. After 3 days of darkness, *atg5* and *atg7* lines showed accelerated senescence characterized by premature chlorosis (**Figure 2B**), and decreased values of the photochemical efficiency of PSII (ΦPSII) and Fv/Fm statistically significative for *atg5* indicating damage of the photosynthetic apparatus (**Figure 2C**). The phenotype observed in darkness was alleviated but not reverted when sucrose was added as an external carbon source (**Figure 2B**). On the other hand, after 3 days of nitrogen deficiency (L-N), *atg5* and *atg7* phenotypes did not differ significantly from the control treatment (LN), but both *atg* lines exhibited senescence at the center of the colony in comparison to the wild-type (**Figure 2B**). After 7 days in all nutrient-deficient conditions both mutant lines were affected (**Figures 2B, C**). However, at this time point, the main significant differences in the decline of Fv/Fm and ΦPSII values observed between wild-type and *atg* mutants were under optimal growth conditions and nitrogen deficiency, as the wild-type was also severely affected under darkness (**Figures 2C**, **1C**). Remarkable to mention is that *atg* mutants maintained the nitrogen starvation response as previously observed in the wild-type, characterized by triggering caulonemata development although few and less-developed gametophores with shorter rhizoids were observed (**Figures 2B**, **3**).

Colony expansion measured by the radial spread of the colony was evaluated, and calculated as the ratio between the colony area at each time point (Af) and the initial area (Ai) (**Figure 2F**). Under optimal growth conditions (LN) no ratio differences were observed between genotypes during the 21-day time-course experiment. However, on day 14, when lines were transferred to the nitrogen-deficient conditions (L-N), the wild-type showed a higher growth rate in comparison to the mutants. On the other hand, the prolonged darkness affected similarly the growth rate between the wild-type and the mutants (**Figure 2B**), a response that was slightly delayed when supplemented with sucrose.

Noteworthily, even under optimal growth and LD conditions, *atg* lines exhibited a senescent yellowish pattern at the center of the colony in comparison to the wild-type (**Figure 2B**, LN), which was associated with significant differences in colony fresh weight (**Figure 2D**, left panel) and lower photosynthesis parameters (**Figure 2B**). Senescence and cell death between the *atg* mutants and the wild-type were observed under all stress conditions tested but a different pattern was clearly observed between carbon and nitrogen deficiencies (**Figures 2D, E**). Taken together, although *PpATG5* and *PpATG7* are not essential genes, these results suggest that under both optimal growth or nutrient-deprived conditions, autophagy has an important contribution to *P. patens* growth and development.

### 2.3 Morphological plasticity triggered in *atg5* and *atg7* to sustain protonemata growth under standard growth conditions

**Figure 4:**
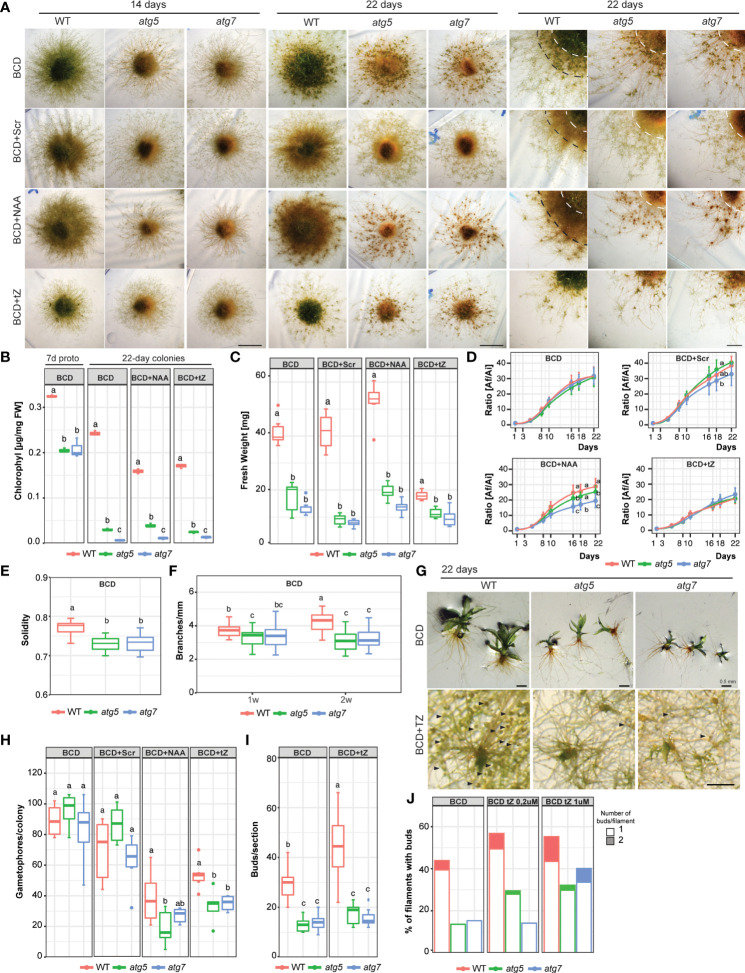
Growth and development changes of *atg* mutants in BCD media, or BCD supplemented with 2% sucrose (BCD+Scr), 1 μM 1-Naphthaleneacetic acid (BCD+NAA) or 0.2 μM trans-Zeatin (BCD+TZ). **(A)** Example images of *P. patens* colonies grown for 14 days (left panel, scale bar: 5 mm), 22 days (middle panel, scale bar: 5 mm), and 22 days with magnification (right panel, scale bar: 2 mm) in BCD media with or without additives. **(B)** Quantification of chlorophyll (μg/mg FW) in 7-day-old protonemata (starting tissue for colonies) and 22-day-old colonies at the end of the experiment. Letters indicate significant differences between genotypes within the same treatment (*n*= 3, One-way ANOVA and Tukey’s HSD test; *P*< 0.01). **(C)** Quantification of colony fresh weight (mg) at the end of the experiment (day 22). Letters indicate significant differences between genotypes within the same treatment (*n*= 6, One-way ANOVA and Tukey’s HSD test; *P*< 0.001). **(D)** Quantification of plant growth during the 22-day-old experiment was calculated as the ratio between the colony area at each time point (Af) and the initial colony area (Ai), under different media. Letters indicate significant differences between genotypes for each time-point (*n*= 9, One-way ANOVA and Tukey’s HSD test; *P*< 0.05). **(E)** Solidity values, defined as area/convex hull area (a value approaching one means the colony has a higher density). Letters indicate significant differences between genotypes in BCD media (*n*= 6, One-way ANOVA and Tukey’s HSD test; *P*< 0.01). **(F)** Quantification of branches per mm of filament in 1- or 2-week old colonies in BCD. Letters indicate significant differences between genotypes and time (*n*= 25, Two-way ANOVA and Tukey’s HSD test; *P*< 0.05). **(G)** Example images of *P. patens* 22*-*day old gametophores in BCD media, and buds (denoted by black arrows) induced by 0.2 μM TZ. **(H)** Quantification of gametophore number per colony after the 22-day growth period. Letters indicate significant differences between genotypes within the same treatment (*n*= 6, One-way ANOVA and Tukey’s HSD test; *P*< 0.05). **(I)** Quantification of buds per section (14 mm^2^) in BCD or BCD supplemented with 0.2 μM trans-Zeatin. Letters indicate significant differences between genotypes and Media (*n*= 8-12, Two-way ANOVA and Tukey’s HSD test; *P*< 0.05). **(J)** Bud quantification per filament in 1-week-old protonemata in BCD or BCD supplemented with 0.2 μM or 1 μM trans-Zeatin, n=60. Values represent the mean ± s.d. of the biological replicates.

To further characterize the effects of autophagy deficiency on *P. patens* growth and development, the wild-type and *atg* mutants were grown on BCD media. This media has nitrate as a single source of nitrogen and favors the transition from chloronemata to caulonemata, and from the juvenile to the adult gametophytic phase allowing the observation of these two distinct developmental processes in more detail. After 14 days of growth phenotypical differences between genotypes were observed, which were exacerbated after 21 days (**Figure 4A**). *atg* mutants were characterized by an early senescence phenotype with low chlorophyll content (**Figure 4B**) and reduced colony weight (**Figure 4C**). Colony expansion did not vary between genotypes in BCD nor under BCD with external addition of sucrose (2%), and cytokinin (0.2 μM TZ) (**Figure 4D**), but significant differences were observed at the end of the treatment with auxin (1 μM NAA). In addition, knock-out lines exhibited a reduction in protonemata density, which was also observed when plants were grown in media supplemented with sucrose or auxin, conditions known to stimulate caulonemata development (**Figure 4A** right panel). This difference in tissue density is reflected in a lower solidity index (**Figure 4E**) and a reduction in the number of branches per millimeter of primary filament (**Figure 4F**) in *atg* mutants. Although a similar number of gametophores were developed in all genotypes in BCD media or BCD supplemented with sucrose (**Figure 4H**), those from *atg* mutants were smaller and those at the central older part of the colony senescence prematurely (**Figure 4G**). Interestingly, the knock-out lines also show an altered response to cytokinin. The addition of 0,2 μM trans-Zeatin (tZ), a low concentration known to induce bud formation, did not affect the number of buds observed in 22-day-old colonies of *atg* mutants (**Figure 4I**). This lack of response could be owed to a failure in cytokinin signal transduction pathway or a consequence of the senescent tissue in these genotypes, which is no longer able to produce new buds. To address this question, young protonemata were cultured with two different tZ concentrations. In *atg5*, the percentage of filaments with buds slightly changed when 0,2 μM tZ was added and no further induction was found when tissue was grown with 1 μM tZ. Meanwhile, *atg7* only responded to the higher tZ concentration (**Figure 4J**). These results indicate that there is, in fact, an alteration in cytokinin response in *atg* mutants.

**Figure 5:**
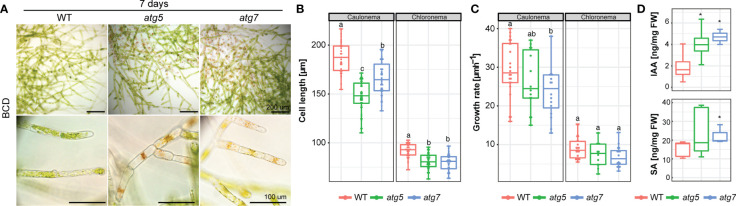
**(A)** Example images of 7-day old *P. patens* protonemata. Scale bar: Upper and middle panels 200 μM, the lower panel 100 μm. **(B)** Quantification of cell length of 7-day old chloronema (*n*=44 wt, *n*= 48 *atg5*, *n*= 42 *atg7*) and caulonema cells (*n*=35 wt, *n*= 51 *atg5*, *n*= 46 *atg7*). Letters indicate significant differences between genotypes for each cell type (One-way ANOVA and Tukey’s HSD test; *P*< 0.05). **(C)** Quantification of the growth rate of 7-day old chloronema (*n*=22 wt, *n*= 12 *atg5*, *n*= 16 *atg7*) and caulonema cells (*n*=22 wt, *n*= 14 *atg5*, *n*= 16 *atg7*). Letters indicate significant differences between genotypes for each cell type (One-way ANOVA and Tukey’s HSD test; *P*< 0.05). **(D)** Quantification of auxin (Indole-3-acetic acid; IAA) and salicylic acid (SA) from wild-type, *atg5* and *atg7* mutants from 7-day old protonemata. *, *P*<0.1 (by Student’s t-test, as compared with the wild type). Values represent the mean ± s.d. of the biological replicates.

The observed differences in protonemata lead us to follow thoroughly the growth of this tissue. Under standard growth conditions (BCD), senescence of the juvenile protonemata could be already distinguished in the distal older regions of 7-day-old protonemata filaments of *atg* mutants, whereas no signs of cell death were observed in the wild-type (**Figure 5A**). Calcofluor staining was used to visualize cell walls and distinguish between chloronema and caulonema cells whose walls are perpendicular or oblique, respectively, to the growth axis. Both *atg5* and *atg7* lines showed a significant reduction in chloronema cell size of 12% and 15% respectively, in comparison to the wild-type, and a reduction of 11% and 14% in *atg5* and *atg7* respectively, was observed in caulonema cell size in comparison to the wild-type (**Figure 5B**). Next, the growth rate of apical tip-growing chloronema and caulonema cells was measured and a significant reduction in caulonema apical cell growth rate but not in chloronema was observed (**Figure 5C**).

The early senescence phenotype together with the protonemata growth response observed, prompted us to measure the auxin (IAA), salicylic acid (SA), abscisic acid (ABA), and cytokinins (CKs) levels in the wild-type and *atg* mutants of 7-day-old protonemata grown in BCD media by LC-MS/MS (**Figure 5D**). Interestingly, both *atg* lines exhibited significantly elevated levels of IAA in comparison to the wild-type. In addition, levels for SA were higher for both *atg* mutants but only statistically significant for *atg*7, whereas ABA and CKs were not detected in the *atg* mutants nor in the wild-type.

Overall, the results suggest that lack of autophagy favors protonemata growth at the expense of a reduction in bud number and gametophore development, thus delaying the adult phase of the moss life cycle and altering the 2D and 3D growth and development. Thus, the spread of the colony is prioritized in *atg* mutants, which agrees with the investment of growth in surface area for nutrient acquisition, although cell density is diminished in addition to a reduction of protonemata cell length and caulonema cell growth rate, a cell type characterized by few chloroplasts and high growth rate in comparison to chloronemata, suggesting that autophagy has a key role in the regulation of growth and development, probably through its hub function in the primary and energetic metabolisms.

### 2.4 *PpATG8a-f* genes are differentially expressed in protonemata under carbon and nitrogen starvation

**Figure 6:**
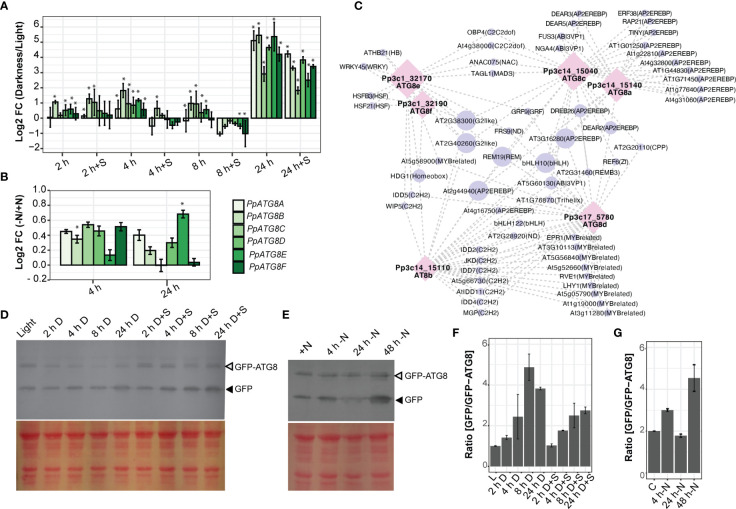
Autophagy is induced under carbon and nitrogen deficiencies. **(A-D)** Expression analysis of *PpATG8s* (*a-f*) in *P. patens* protonemata. Log_2_-fold change values determined by qPCR upon darkness treatment or darkness supplemented with 2% Sucrose after 2 h, 4 h, 8 h, or 24 h, compared to 16 h light treatment **(A)**, or upon nitrogen deficiency treatment after 4 h or 24 h versus optimal nitrogen conditions **(B)**. Error bars depict the standard error of the mean (n=3). *, *P*<0.05 (by two-tailed and paired Student’s t-test) between treatment against controls (16 h light for darkness analysis and full nitrogen for Nitrogen deficiency, respectively. **(C)** Gene–motif interaction network of *PpATG8s* genes. Motifs were filtered by a motif Score > 9 and with at least two degrees (**Tables S4**, **S5**). Nodes are represented as *PpATG8s* transcripts (purple circle) and motifs (pink diamond), also their size displays the number of connections. Edges show their interaction (gray dash line). **(D, E)** Autophagic flux analysis in *PpATG8e::GFP-PpATG8e* moss protonemata by the GFP-ATG8 processing assay. **(D)** Upper panel: Anti-GFP immunoblot was performed using protein extracts of 7-day old protonemata of *PpATG8e::GFP-PpATG8e* line under optimal growth conditions (16h light, L), or after 2h, 4h, 8h, or 24 h treatment of darkness or darkness supplemented with 2% Sucrose (D+S). Lower panel: Protein loading control stained with Red Ponceau. **(E)** Upper panel: Anti-GFP immunoblot performed using protein extracts of 7-day old protonemata of *PpATG8e::GFP-PpATG8e* line under continuous light and optimal nitrogen conditions (+N), or after treatment of nitrogen-deficient medium (-N) for 4h, 24h, and 48h. Lower panel: Protein loading control stained with Red Ponceau. Quantification of the protein bands shown in **(F)** and **(G)**, respectively. The intensity of bands corresponding to free GFP moiety (black arrowheads) normalized by the intensity of full-length GFP-ATG8 bands (grey arrowheads) is plotted (mean ± SE; n = 2).

The early senescence pattern observed in the juvenile protonemata of *atg* mutants motivated us to further analyze the expression pattern of *P. patens* genes coding for the ubiquitin-like protein *ATG8* family (*PpATG8a-f*), involved in the early steps of autophagy induction. All *PpATG8s* belong to the Clade I *ATG8s* (**Figure S1**), with an absence of members in Clade II. Gene expression was evaluated by real-time RT-PCR (**Figures 6A, B**) at different time points during the night of a LD photoperiod (2 h, 4 h, and 8 h), and extended darkness (24 h) compared to 16h light (control), and during nitrogen starvation compared to optimal nitrogen content under continuous light. The expression levels of all *PpATG8s* were rapidly induced during the first 2 h of darkness, peaked after 4 h of darkness, and by the end of the night (8 h of darkness) declined close to the levels observed at the first 2 h of darkness (**Figure 6A**), being the most responsive genes *PpATG8b*, *PpATG8c*, and *PpATG8e*. During darkness supplemented with sucrose (2% w/v), *PpATG8b* and *PpATG8c* were upregulated after 2 h, whereas all *PpATG8s* were downregulated at 4 h and 8 h of darkness, except for *PpATG8b* which was still upregulated at 4 h. Interestingly, under extended darkness (24 h) independent of the presence of an external carbon source all *PpATG8s* were strongly upregulated, although higher expression levels were observed in the absence of sucrose. Upon nitrogen starvation, almost all *PpATG8s* were upregulated. Expression levels were higher at 4 h than at 24 h except for *PpATG8e* which achieved the highest expression levels at 24 h (**Figure 6B**). Taken together, although *PpATG8a-f* genes were upregulated under darkness and nitrogen starvation, subtle changes in their expression signature were observed between both conditions, namely darkness elicited higher fold changes. In addition, *PpATG8s* expression was upregulated even with an external carbon source suggesting a strong regulation by light.

A search for cis-acting regulatory elements present in the *PpATG8s* promoter regions showed that the six *PpATG8* promoters are enriched in light and stress response elements compared to the reference gene *PpADE-PRT* (**Figure S4**) and five of them (*PpATG8b-f*) have a higher number of hormone response elements. Most of the elements involved in light, ABA, auxin, and abiotic stress responses are absent in the control gene, suggesting that these conditions regulate *PpATG8* gene expression in *P. patens*. Next, a transcription factor (TF) survey of binding sites (BS) was performed on the *PpATG8a-f* gene promoters using the Homer platform (**Tables S4, S5**). Several TFBS were present in promoters from a subset of *PpATG8s*, suggesting common regulatory features (**Figure 6C**). As an example, *PpATG8a* and *PpATG8c* shared several TFBS associated mainly with abiotic stress responses such as AP2/EREBP most of them belonging to drought response (DREB type), *PpATG8e* and *PpATG8f* shared sites for HSF21 and HSFB3 involved in the heat shock response in addition to a WRKY45 BS, a TF highly induced during both developmental and dark‐induced senescence recently shown to be involved in the plant metabolic reprogramming following carbon starvation (Barros et al., 2022). Interestingly, sites identified in the promoter region of various members from *PpATG8a-f* are involved in growth and development such as bHLHs and C2H2 several associated with Arabidopsis root development, and MYB TFs are involved in the regulation of circadian rhythms such as RVE7, RVE6, RVE1.

### 2.5 Monitoring autophagy in protonemata under carbon and nitrogen starvation through a *PpATG8e::GFP-PpATG8e* moss reporter line

Our gene expression analysis showed that *PpATG8e* (*Pp3c1_32170V3.1)* was one of the highly responsive *PpATG8* family genes to carbon and nitrogen starvation in protonemata (**Figures 6A, B**). Thus, to monitor autophagy induction under these conditions the *PpATG8e::GFP-PpATG8e* reporter line (Sanchez-Vera et al., 2017) was used to measure the autophagic flux through the GFP-ATG8 processing assay. This assay is based on the accumulation of free-GFP derived from the GFP-ATG8e fusion due to the relatively high stability of the GFP moiety once inside the vacuole, which can be detected and quantified by immunoblot analysis with anti-GFP antibodies (Chung et al., 2010).

Protein extracts from seven-day-old protonemata under optimal growth conditions (LD photoperiod) were collected after 16 h of light, or at different time points (2 h, 4 h, 8 h, and 24 h) during darkness or darkness supplemented with 2% sucrose as an external carbon source. As observed in the immunoblot (**Figures 6D, F**) and subsequent quantification, the ratio of [GFP/GFP-PpATG8e] increases during the period of darkness with the highest value at 8 h of darkness. Similarly, but to a lower extent, autophagy flux was still induced in darkness with sucrose, with increasing values as long as the dark period extended achieving similar levels at 8 and 24 h. As performed for gene expression analysis, measurements of autophagic flux under nitrogen starvation conditions were performed under continuous light, and the results indicate that autophagy flux increases at 4 h and 48 h of N starvations but decreases at 24 h (**Figures 6E, G**).

### 2.6 Autophagy is differentially induced in apical growing protonemata

The TFBS survey showed that several *ATG* genes associate with TFs involved in developmental processes and reprogramming events (**Figure 6C**). This led us to analyze *PpATG4a-b*, *PpATG5*, *PpATG7*, and *PpATG8a-f* expression data from a leaflet detachment experiment in which individual leaflet cells undergo reprogramming and become an apical stem cell, consecutively generating a new protonema filament (Busch et al., 2013). Reprogramming events take place between 6 h and 36 h after a leaflet is detached, matching with an increase in *PpFIE* expression, a reference gene used to indicate this developmental process. First protonemal filaments start developing 48 h to 72 h after the incision, thus indicating the onset of apical growth events. **Figure S5A** shows that *PpATG8a*, *PpATG8b*, *PpATG8e*, *PpATG4b, PpATG5*, and *PpATG7* increased their expression during the first hours after detachment, as expected for stress-induced genes and as previously reported to be a signature of autophagy during somatic reprogramming (Kanne et al., 2021). In addition, the expression of almost all *ATG* genes analyzed also increased in the 48 h to 72 h time window after the incision, providing initial evidence that supports a link between autophagy and the development of the polar growing protonemata. Furthermore, the expression data for this tissue grown in optimal conditions (Xiao et al., 2011; Agudelo-Romero et al., 2020) indicated that levels of most *ATG* genes are higher in caulonemata than in chloronemata (**Figure S5B**). Considering that caulonemata elongate three times faster than chloronemata, these observations suggest a role for autophagy in apical growth.

**Figure 7:**
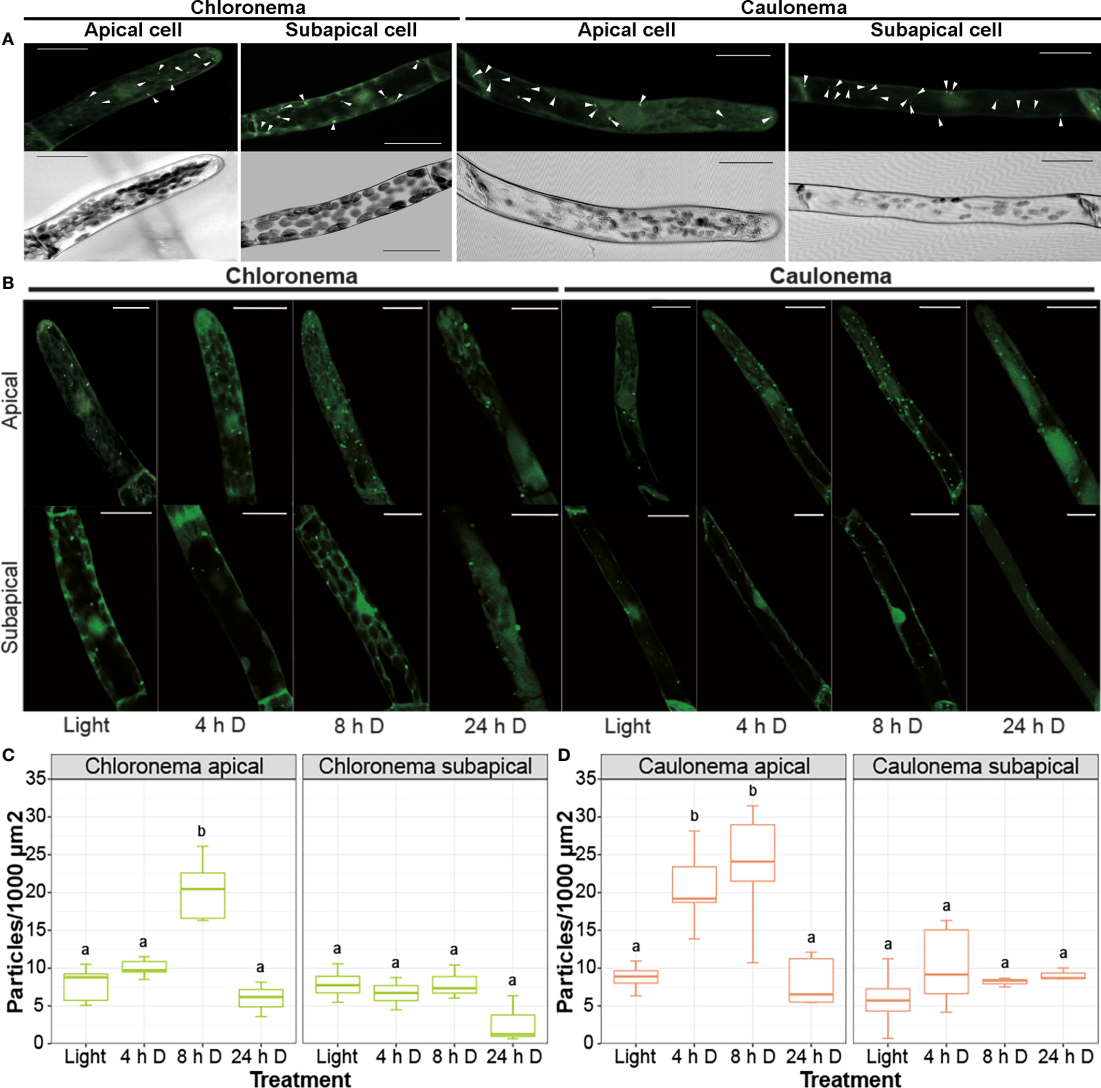
Autophagy is differentially induced in protonemata apical cells during a long day/night cycle. **(A)** Representative images of control (light) protonemata cells expressing *PpATG8e::GFP-PpATG8e.* Upper panels show the fluorescence pattern of *PpATG8e::GFP-PpATG8e*, with autophagic particles (bright dots) indicated by white arrowheads; lower panels show the corresponding trans image of each cell. **(B)** Representative images of protonemata cells expressing *PpATG8e::GFP-PpATG8e* under optimal growth conditions (light, 4h and 8h darkness) and extended darkness (24h darkness). Final images were obtained from z-stacks of 4-5 confocal planes. Scale bars: 20 μm. **(C, D)** Quantification of autophagic particles in chloronema **(C)** and caulonema **(D)** apical and subapical cells. The number of particles was quantified per single cell area and then referred to 1000 μm^2^. Values represent the mean ± s.d. of the biological replicates. A separate statistical analysis was performed for each cell group (chloronema apical, chloronema subapical, caulonema apical, caulonema subapical). Letters indicate groups with significantly different means (n= 7-10; One-way ANOVA and Tukey’s HSD *post hoc* test; *P* < 0.05).

To get further insight into the potential connection between these processes, the number of autophagic vesicles in apical and subapical protonemata cells expressing *PpATG8e::GFP-PpATG8e* was quantified during the dark hours of a LD photoperiod under optimal growth conditions. In chloronema apical cells, the number of autophagic particles significantly increased after 8 h of darkness, that is by the end of the dark hours in a LD photoperiod (**Figures 7A, B**). However, in caulonemata, apical cells showed a significant increase in GFP-PpATG8e vesicles earlier during the night, at 4 h and 8 h (**Figures 7A, C**). No significant differences were observed in subapical cells in any of the cases. These results suggest that autophagy could sustain the apical growth of protonemata cells during darkness under optimal growth conditions, probably acting as an energy source during the night to sustain caulonemata growth. The addition of LY294002, a PI3K inhibitor, prevented the formation of GFP-PpATG8e vesicles in light and diminished their accumulation during the night, confirming that the observed puncta are, in fact, autophagosomes (**Figure S6**). The autophagic vesicles upon 24 h of extended darkness were also quantified. In this case, the number of particles remained similar to the control situation (light) in every analyzed cell (**Figures 7B, C**).

**Figure 8:**
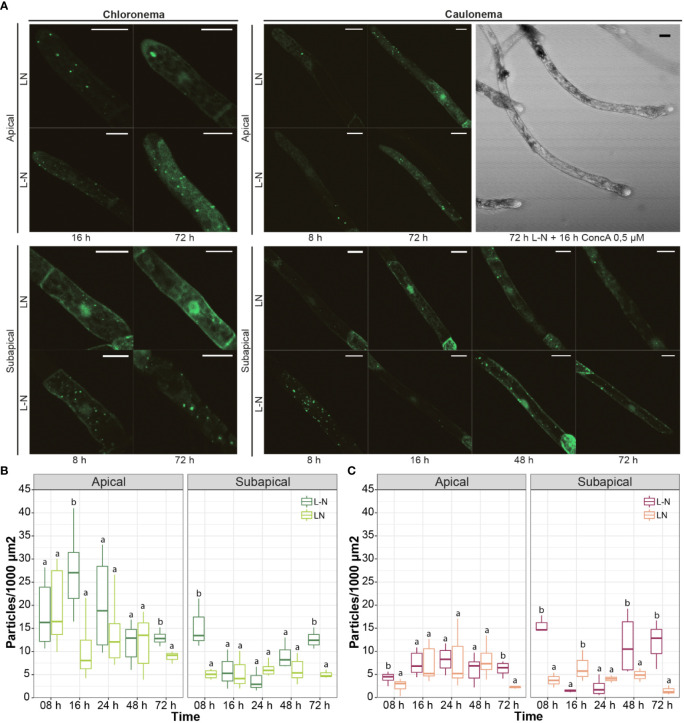
Autophagic response of protonemata cells to nitrogen deficiency. **(A)** Representative images of protonemata cells expressing *PpATG8e::GFP-PpATG8e* under optimal growth conditions (LN, BCDAT medium) and nitrogen deficiency (L-N, BCD/NaK medium; 8h, 16h, 24h, 48h, and 72h). Images shown correspond to time points that were significantly different from the controls. Cells were kept in continuous light to avoid the effect of darkness. Final images were obtained from z-stacks of 4-5 confocal planes. Scale bars: 20 μm. **(B, C)** Quantification of autophagic particles in chloronema **(B)** and caulonema **(C)** apical and subapical cells. The number of particles was quantified per single cell area and then referred to 1000 μm^2^. Values represent the mean ± s.d. of the biological replicates. For each cell group (chloronema apical, chloronema subapical, caulonema apical, caulonema subapical), a separate statistical analysis was performed on every time point comparing the control (BCDAT) with the treatment (BCD/NaK). Letters indicate groups with significantly different means (n= 7-10; One-way ANOVA and Tukey’s HSD *post hoc* test; *P* < 0.05).

Next, the autophagic response of protonemata cells under nitrogen deficiency was analyzed. Concanamycin A (ConcA) is commonly used to study the accumulation of autophagic bodies under nitrogen deficiency, because it reduces the acidification of the vacuole and consequently inactivates vacuolar lytic enzymes. This is particularly relevant in experiments performed in light since darkness also triggers an inhibitory effect on H+ ATPase activity (Tamura et al., 2003). After exploring protonemata response to different concentrations and incubation times, cells were incubated with ConcA 0.5 μM for 16 h. Longer caulonema cells showed tip swelling and expulsion of intracellular material (**Figure 8A**: caulonema, upper right panel). As DMSO-treated cells grew normally, this might be a cytotoxic effect of ConcA *per se*. Lowering ConcA concentration to 0.25 μM prevented cell damage but no significant differences were observed in treatments with or without the drug (data not shown). Due to these negative effects, quantification of autophagic vesicles under nitrogen deficit was performed without ConcA.

As in *Arabidopsis*, in which autophagic induction by nitrogen deficiency is known to occur later than the one induced by darkness (Liu et al., 2020) a solid increase in GFP-PpATG8e vesicles was observed after 72 h of L-N treatment in both protonemal cell types, including apical and subapical cells (**Figures 8A-C**). **Figure 8** also shows that in apical cells, particles increased significantly at 8 h L-N in caulonemata and 16 h L-N in chloronemata. However, after these time points, differences with the control became slighter in both cases until 72 h of deficiency were reached. This result reinforces the idea of autophagy triggering earlier in caulonemata apical cells to sustain their higher growth compared to chloronemata. A major difference between these responses and the ones induced by darkness is that subapical cells increased the number of autophagic particles under nitrogen starvation. The induction took place at 8 h and 72 h L-N in chloronemata and 8 h, 48 h, and 72 h in caulonemata. This suggests a different role for autophagy in nitrogen starvation conditions, possibly remobilizing the nutrient from subapical to apical cells to allow the latter to continue their growth.

## 3 Discussion

This study primarily aimed to determine whether autophagy plays a role in moss protonemata apical growth and hence development, in the context of the multicellular tissue of the bryophyte *P. patens*. Although it was shown that *atg5* mutants were hypersensitive to carbon and nitrogen starvation (Mukae et al., 2015). our work expands the knowledge on this response under both optimal and nutrient-deprived conditions through a deep phenotypic characterization of *atg5* and *atg7* mutants combined with methods to monitor autophagy. Our results provide evidence indicating that autophagy has roles during apical growth with differential responses within the cell types of the same tissue and contributes to life cycle progression and thus to the 2D and 3D growth and development of *P. patens.*

### 3.1 All core ATG proteins are present in *P. patens* with the six ubiquitin-like proteins PpATG8a-f belonging to Clade I

Our data showed that all the core *ATG* genes are present in *P. patens*. Nonetheless, specific gene families such as *PpATG8* and *PpATG18* are reduced in comparison with *A. thaliana*, but enriched compared to algae and other bryophytes such as liverworts or hornworts (Norizuki et al., 2019), which is probably the result of two ancestral whole-genome duplications (WGD) events in *P. patens* (Lang et al., 2018). Different from previous reports, our search for *ATG8s* was enriched in species from Pyramimonadophyceae, Chlorophyceae, Chlorokybophyceae, Klebsormidiophyceae, Charophyceae, Zygnemophyceae algae, and bryophytes. All the algae species contain a single-copy *ATG8* except for the single-celled *Penium margaritaceum*, a member of the Zygnematophyceae, the closest relatives of land plants, which has 2 copies. Although *P. margaritaceum* has not undergone any recent WGD, it has expanded repertoires of gene families, signaling networks, and stress responses that are associated with terrestrialization, which is in consonance with the roles of autophagy in the adaptation of plants to stress (Jiao et al., 2020).

The phylogenetic analysis resulted in two different ATG8 clades, confirming previous reports; Clade I including most of the Viridiplantae ATG8 members including all algae and bryophyte ATG8s, and clade II exclusively found in Ferns, Gymnosperms, and Angiosperms (Seo et al., 2016; Kellner et al., 2017; Bu et al., 2020). Interestingly, our results showed that several Clade I ATG8s belonging to bryophytes and streptophyte algae but not chlorophyte algae lack the extra amino acid residues at the C-terminus after the glycine residue, previously found only in some Clade II ATG8s, such as AtATG8h-i, that allows interaction with the autophagosome membrane without ATG4 processing (Seo et al., 2016). Thus, analyses of the different Clade I and II ATG8s will be necessary to compare their requirements for ATG4 processing and to further understand their specific functions.

### 3.2 The protective and antisenescence role of autophagy to the canonical inducers carbon and nitrogen deficiencies is conserved in *P. patens*

Carbon and nitrogen starvation are canonical inducers of senescence and autophagy in plant cells. Carbon starvation-induced in response to light deprivation had a more severe effect than nitrogen deficiency in the growth and development of wild-type *P. patens*, inducing a marked rapid senescence phenotype characterized by a visible yellowing due to damage of the photosynthetic apparatus and chlorophyll degradation leading to the cessation of protonemata growth and gametophore development. Similar physiological responses to dark-induced senescence were widely described in many plants (Buchanan-Wollaston et al., 2005; Liebsch and Keech, 2016; Paluch-Lubawa et al., 2021). Moss *atg5* and *atg7* mutants showed accelerated senescence, which was mainly exacerbated under carbon starvation, where chlorophylls, photosynthesis, and weight were decreased and cell death increased, drastically in comparison to wild-type. The accelerated senescence of *A. thaliana atg* mutants has been related to the increment in ROS generation and SA levels (Yoshimoto et al., 2009; Masclaux-Daubresse et al., 2014), and an increase in SA was observed in *P. patens* although only statistically significant in *atg7*.

The symptoms caused by nitrogen deficiency in wild-type *P. patens* were more related to the induction of starvation avoidance and survival strategies, promoting the growth of caulonemata and rhizoids, and these strategies were not abolished in the *atg* mutants. Moss rhizoids though multicellular are analogous to root hairs, apical growing cells providing nutrition and anchorage to the substrate (Menand et al., 2007b). The stimulation of rhizoid growth of wild-type *P. patens* under nitrogen deficiency resembles Arabidopsis root hair growth triggered under low-nitrate conditions (Vatter et al., 2015). Although moss *atg* mutants maintained the development of caulonemata and rhizoids, final caulonemata cell length and rhizoids were shorter, and growth was sustained at the expense of the early senescence of the central part of the colony, by reducing gametophore size (see discussion below).

### 3.3 Autophagy contributes to the *P. patens* 2D and 3D growth and developmental program and its absence favors protonemata growth (2D) at the expense of the adult gametophytic phase (3D)

Arabidopsis *atg* mutants, with exception of those genes involved in the PI3K complex (PI3K, VPS15, ATG6), when grown under optimal growth conditions and LD photoperiod germinated and developed as wild-type plants (Izumi et al., 2013). However, under these conditions, *P. patens atg* mutants exhibited several defects in growth and development in gametophores and protonemata, such as stunted gametophores, premature senescence, hyposensitivity to cytokinin that exert an antisenescence effect through primary metabolism, and a dampened response to sucrose and auxin, which are known to stimulate caulonemata growth. Moreover, *atg5* and *atg7* did not show defects to differentiate chloronemal and caulonemal tip-growing cells, albeit differences such as reduced chloronema and caulonema cell length and reduced apical caulonemata growth rate were observed, suggesting that autophagy has a role sustaining their apical growth. The role of autophagy in plant polar growth has almost been underexplored, with a study showing that PTEN, a phosphatase of PI3P regulates autophagy in pollen tubes (Zhang et al., 2011), and another evidencing that *PI3K* loss-of-function in common bean plants have shorter root hairs and severe affections in the infection thread (Estrada-Navarrete et al., 2016). In addition, caulonema tip growth is reminiscent of fungal hyphae (Bartoszewska and Kiel, 2011) since the *atg5* and *atg7* caulonemata growth could be compared with the response of the *atg1* line of *Aspergillus fumigatus* which, also resulted in inhibition of colony growth under starvation (Richie et al., 2007). These facts revealed the physiological importance of autophagy for polar tip growth of filamentous tissue in charge of nutrient acquisition and its adaptive role to cope with starvation. Interestingly, new evidence has provided insights supporting the notion that autophagy may have effects on vacuolar enlargement, which in turn could impact cell elongation (Robert et al., 2021a; Robert et al., 2021b).

Nevertheless, under all conditions tested, it was revealed a clear strategy of *atg* mutants at the whole plant level, where protonemata growth is favored at the expense of gametophore development and thus life cycle progression. The higher levels of auxin measured in protonemata in *atg* mutants suggest that the signal to trigger the differentiation of more caulonemata cells is present, which was not translated into an increase in the number of this cell type, probably preventing unaffordable growth according to the moss energetic resources but maintain caulonemata differentiation. Other mutants impaired in energy signaling such as the double knock-out of trehalose-6-phosphate synthase (*tps1-tps2*), and the hexokinase-1 (*hxk1*), are negatively affected in caulonemata differentiation (Olsson et al., 2003; Phan et al., 2020). Moreover, the phenotypes of the energy sensor Snf1-related protein kinase 1 (*snf1a-snf1b*), known to activate autophagy and inhibit the TOR-kinase complex in Arabidopsis, shared several phenotypes in common with the moss *atg5* and *atg7* lines such as premature senescence, few leafy shoots with shorter stems and smaller leaves, hyposensitivity to cytokinins, and shorter chloronemata cells (Thelander et al., 2004). However, the *snf1a-snf1b* exhibited an excess of caulonemata due to an increased sensitivity to auxin, few aberrant chloronemal filaments, and failed to grow in a LD photoperiod; which are severe phenotypes in comparison to the *atg* mutants possibly due to the fact that SNRK1 is upstream autophagy, and integrated signals from multiple pathways.

### 3.4 Autophagy is differentially induced in moss protonemata by carbon or nitrogen starvation

Gene expression analysis under carbon starvation during the night of a LD photoperiod, and during extended darkness (24 h), with and without sucrose allowed to study the effect of light on autophagy. A general trend observed for several *PpATG8s* genes was their up-regulation with the shift from 16h light to darkness during the first 4 h of darkness. At 8 h of darkness, albeit their expression levels were still up-regulated they diminished closer to the 2 h darkness time point. Similar behavior was reported for *ATG6*, *PI3K*, *ATG7*, and *ATG9* in Arabidopsis during the night, associated with leaf starch degradation by autophagy (Wang et al., 2013). Interestingly, our *PpATG8s* promoter analysis showed several TFBs involved in the regulation of circadian rhythms. After extended darkness (24 h) the levels of all genes were up-regulated reaching the highest levels of expression in the medium with or without sucrose, strongly suggesting the role of light in autophagy induction, consistent with previous observations in Arabidopsis showing that light signaling could regulate autophagy through modulating the transcription level of *ATGs* (Yang et al., 2020).

Another approach used to study the autophagic response in protonemata was the quantification of autophagic particles in the *PpATG8e::GFP-PpATG8*e reporter line. The observation of cells during the night (4 h and 8 h darkness) revealed a differential response depending on the cell type and the position of the cell along the protonemal filament. Apical cells, which are actively growing, increased the number of GFP-PpATG8e particles during the night. Interestingly, earlier increases were observed in caulonemata compared to chloronemata. On the other hand, the basal number of autophagic vesicles in subapical cells, in which growth has ceased, did not change during the dark hours of the photoperiod.

In vascular plants, autophagy contributes to the increase in energy supply through the degradation of stromal proteins into free amino acids, RCB pathway (Ishida et al., 2008) or starch granules breakdown, SSGL body pathway (Wang et al., 2013). Considering the higher growth rate and the lower number of chloroplasts in caulonema apical cells compared to chloronema, our observations support the idea of autophagy triggering earlier during the night in caulonemata as an energy-providing mechanism to sustain the growth processes in the dark. These results are consistent with the above-mentioned phenotype reflecting impaired energetic resources in *atg* mutants. It is plausible that different components of the cellular machinery are the cargo for these distinct cell types, which needs to be further addressed.

Our qPCR data and autophagic flux analysis indicate that autophagy is activated in extended darkness (24 h) but the number of GFP-PpATG8e particles appears to stay the same as in the control situation. This may be due to an enhanced autophagic flux rapidly delivering vesicles to the vacuole for degradation, as suggested by the GFP fluorescence inside this organelle, or to free GFP accumulation in vacuoles under dark conditions, which increases fluorescence making it difficult to distinguish autophagic bodies in the lumen.

In tissue grown in light without nitrogen (L-N), the time difference in autophagic particles accumulation between chloronema and caulonema apical cells was still observed, but a different response was detected in subapical cells compared to darkness. In this case, subapical cells of both protonemata cell types increased the number of autophagic particles at different time points. These observations are compatible with the previously described role of autophagy in nitrogen remobilization during senescence and nitrogen starvation in vascular plants (Guiboileau et al., 2012; Li et al., 2015). Our observations showed that in the *atg* mutants older cells of a filament died prematurely, suggesting that another mechanism than autophagy could be involved in recycling components of older cells to sustain protonemata apical growth. During energy starvation in Arabidopsis, an autophagic-independent route of chloroplast degradation associated with the Chloroplast vesiculation (CV) pathway is highly induced in the absence of autophagy contributing to the early senescence phenotype of *atg* mutants (Wang and Blumwald, 2014). However, we did not find homologs of the CV gene (AT2G25625) neither in algae nor in bryophytes genomes.

### 3.5 Model summarizing the effects of autophagy deficiency in *Physcomitrium patens*

**Figure 9:**
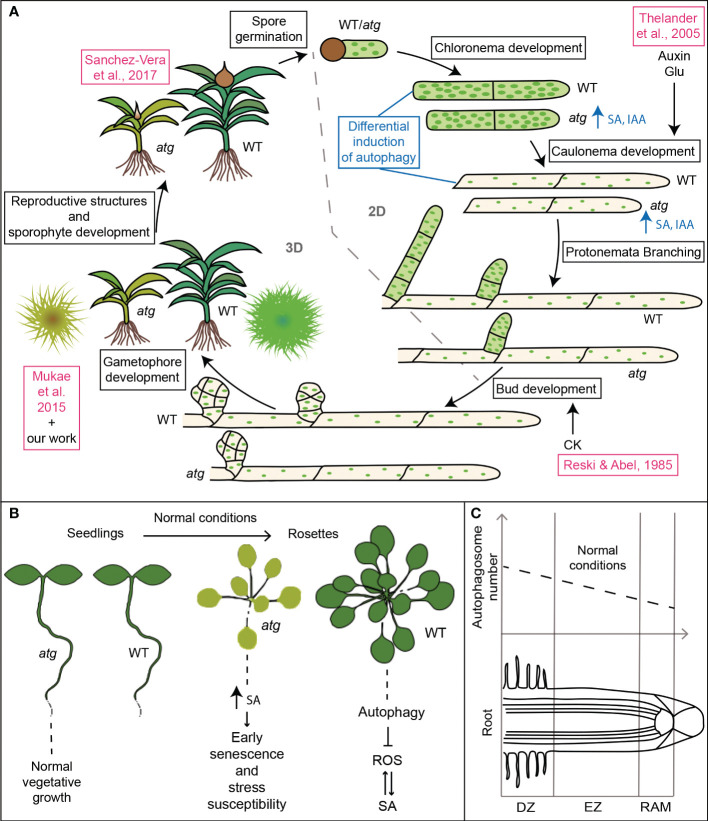
Roles of autophagy in plant development. **(A)** Model summarizing the effects of autophagy deficiency in *Physcomitrium patens* life cycle. Features in blue indicate additional results that support the changes in growth and development described in the scheme. Features in pink indicate previously published data in *P. patens* (Reski and Abel, 1985; Mukae et al., 2015); Sanchez-Vera et al., 2017); (Thelander et al., 2005). **(B, C)** Comparative data regarding the role of autophagy in *Arabidopsis thaliana* development. **(B)** Plants grown on nutrient rich soil (Doelling et al., 2002; Yoshimoto et al., 2004; Yoshimoto et al., 2009; Masclaux-Daubresse et al., 2014). **(C)** Basal autophagic activity in the different root zones (Inoue et al., 2006; Yano et al., 2007). DZ, differentiation zone; EZ, elongation zone; RAM, root apical meristem.

Autophagy-deficient moss genotypes presented premature senescence and hypersensitivity to nutrient starvation as previously reported (Mukae et al., 2015), and similar to the phenotype observed in *atg* mutants in *Arabidopsis* (Doelling et al., 2002; Yoshimoto et al., 2004); **Figure 9B**). Besides, we showed here that autophagy mutants in *P. patens* have higher levels of SA than wild type (**Figures 5D**, **9A**), thus suggesting that autophagy-mediated senescence in the moss might be dependent on the SA pathway, as reported in *Arabidopsis* (Yoshimoto et al., 2009; Masclaux-Daubresse et al., 2014) (**Figure 9B**). Furthermore, the increased level of IAA observed in *atg* mutants in *P. patens* also provided evidence suggesting a conserved interplay between autophagy and hormones in the moss (**Figures 5D**, **9A**). Further investigations are needed to unravel how the *atg* phenotype regarding protonemata growth and development is related to auxin levels, energy availability or sugar signaling.

Interestingly, while *atg Arabidopsis* mutant seedlings show normal vegetative growth (**Figure 9B**), autophagy-deficient moss genotypes showed altered vegetative growth and development, as indicated by the differential effects on the protonemata cells, chloronema and caulonema (**Figure 9A**). Autophagy deficiency favored the 2D growth of the colony through protonemata growth at the expense of a reduction in the 3D growth, affecting the development of buds and gametophore, and thus the adult gametophytic phase. Moreover, as it was reported for other land plants (Norizuki et al., 2020), autophagy deficiency also impairs gamete differentiation in *P. patens*, affecting the reproductive stage of the moss (Sanchez-Vera et al., 2017).

We also reported a differential induction of autophagy during the night of a long-day photoperiod in WT protonemata cells (**Figures 7**, **9A**). Interestingly, under normal conditions, WT *Arabidopsis* roots show different levels of autophagy depending on the root zone (Inoue et al., 2006; Yano et al., 2007) (**Figure 9C**). The highest levels are observed in the differentiation zone, where root hairs are formed. Authors conclude that autophagy is involved in cell growth and differentiation processes, such as root hair formation. Remarkably, protonemata cells and root hairs both depend on apical growth for elongation, supporting the hypothesis of autophagy acting as a mechanism to facilitate this process. In this context, whether autophagy acts as an energy-providing mechanism, or rather as a reprogramming one, still needs to be assessed.

Taken together, our work expands the knowledge of autophagy in bryophytes, has identified several components of the *P. patens* autophagic system, and provides valuable results indicating that autophagy has roles during apical growth highlighting a degree of cell-type specificity in the autophagy response within the same tissue, which in turn contributes to the development of the 2D and 3D tissues of *P. patens*. Although further aspects of this proposed model remain to be demonstrated, it offers additional perspectives on the physiological roles of autophagy in plants, opening new avenues to explore in the future.

## 4 Materials and methods

### 4.1 Identification of *ATG* genes in *P. patens* and phylogenetic analysis

Orthologs *ATG* genes from the moss *P. patens* were retrieved by reciprocal Basic Local Alignment Search Tool for Protein (BLASTP) and Nucleotide (TBLASTN) searches using the coding regions of *ATG* genes previously described in *A. thaliana* performed in Phytozome v12.1 database (https://phytozome.jgi.doe.gov) (**Table S1**). For phylogenetic analyses, a similar search was performed for ATG8 protein sequences from several algae and plant species in Phytozome v12.1 database and ONEKP: BLAST for 1,000 Plants (https://db.cngb.org/onekp/species/) (**Table S2**). Evolutionary analyses were conducted in MEGAX (Kumar et al., 2018). Multiple sequence alignment was inferred using MUSCLE. The evolutionary history was inferred by using the Maximum Likelihood method and JTT matrix-based model and the bootstrap consensus tree was inferred from 1000 replicates.

### 4.2 *PpATG8s* promoter and network analysis

Analysis of transcription factor binding sites (TFBSs) was done using upstream sequence regions between 0.6 kb and 2.5 kb, depending on the flanking gene boundaries according to *P. patens* genome v3.3 (https://phytozome.jgi.doe.gov/pz/portal.html). These sequences were investigated with Homer v4.11.1 software (https://homer.ucsd.edu/homer/motif/), assessing 506 motifs from the Homer plant database against the six *PpATG8s* genes using the parameter “-find” from the homer2 “findMotifs.pl” script. Then, motif score > 9 were selected for downstream analyses (**Table S4**). Gene–motif interaction network was built representing the relationship between gene and known motif employing Cytoscape 3.8.2 software (Shannon et al., 2003). Co-expression network table was generated using “NetworkAnalyzer” tool from Cytoscape, keeping nodes with at least two degrees (**Table S5**).

### 4.3 Plant material, growth conditions

All experiments described in this study, including the generation of P. patens mutant lines, were performed with *Physcomitrium (Physcomitrella) patens ssp. patens* (Hedwig) ecotype ‘Gransden 2004’. Plant cultures were grown axenically at 24°C under a long-day photoperiod (16-h light/8-h dark) with a photon flux of 60-80 μmol.m^2^.s^-1^. *P. patens* protonemal tissue was subcultured routinely at 7-day intervals on cellophane disks (AA packaging) overlaying Petri dishes (90 mm in diameter) that contained BCDAT medium [0.92 g/L di-ammonium tartrate (C_4_H_12_N_2_O_6_), 0.25 g/L MgSO_4_.7H_2_O, 1.01 g/L KNO_3_, 0.0125 g/L FeSO_4_.7H_2_O, 0.25 g/L KH2PO4 (pH 6.5), 0.147 g/L CaCl_2_.2H_2_O, and 0.001% Trace Element Solution (0.055 g/L CuSO_4_.5H_2_O, 0.055 g/L ZnSO_4_.7H_2_O, 0.614 g/L H_3_BO_3_, 0.389 g/L MnCl_2_.4H_2_O, 0.055 g/L CoCl_2_.6H_2_O, 0.028 g/L KI, 0.025 g/L Na_2_MoO_4_.2H_2_O)] and 7 g/L agar, or minimal media BCD where the di-ammonium tartrate from BCDAT media was omitted (Ashton and Cove, 1977).

### 4.4 Generation of *atg5* and *atg7* knock out mutants in *P. patens*

Polyethylene glycol–mediated protoplast transformation was performed according to (Saavedra et al., 2015). In short, 6-day-old protonemata were treated with 0.5% Driselase (Sigma-Aldrich) in 8.5% w/v mannitol for 30 min, passed through a 100-mm sieve, incubated for 15 min at room temperature, and passed through a 50-mm sieve. The protoplasts of the final flow-through, which were washed twice in 8.5% mannitol, were ready for further use. Protoplasts were transformed at a concentration of 1.6·10^6^ protoplasts.ml^-1^. Each transformation consisted of 0.3 ml of protoplast suspension and 10-15 μg of linear DNA. To eliminate any episomal-resistant colonies, two rounds of selection were undertaken using the appropriate antibiotic. Transformations with knock-out vectors were performed with plasmid-based vectors linearized with the appropriate restriction enzyme (Thermo Fisher Scientific).

### 4.5 Starvation, hormonal and pharmacological treatments

For colony experiments in response to starvation, moss colonies were initiated from 1 mm2 spot cultures on cellophane overlaid BCDAT media and grow for 14 days. Then the cellophane was transferred to new media for control (BCDAT), and then kept under optimal growth light conditions (LD photoperiod) either with nitrogen (LN) or without nitrogen supply (L-N), or transferred to darkness (DN) or darkness with an external supplement of sucrose (DN+S), for additional 3 or 7 days. For colony experiments in minimal media (BCD), moss colonies were initiated from 1 mm^2^ spot cultures on cellophane overlaid BCD media without or with additives applied to the media to a final concentration of 1 μM for auxin (1-Naphthylacetic acid, NAA, Sigma-Aldrich), 0.2 μM trans-Zeatin (tZ, Sigma-Aldrich) or 2% (w/v) Sucrose (Sigma-Aldrich). Moss colonies were imaged with an Olympus SZX16 stereomicroscope equipped with a color camera (Olympus DP71).

For cytokinin treatment in protonemata, tissue was grown for 7 days on solid BCD medium without or with 0.2 μM and 1 0.2 μM tZ. Cells were imaged using an Olympus SZX16 stereomicroscope or an Olympus BX-61 microscope, both equipped with an Olympus DP71 color camera. For carbon starvation experiments, protonemata were first grown for 7 days under LD photoperiod (16-h light/8-h dark) on solid BCDAT medium before being transferred to darkness without or supplemented with 2% sucrose during the indicated number of hours (h). For nitrogen starvation experiments, protonemata were first grown for 7 days under LD photoperiod (16-h light/8-h dark) on solid BCDAT medium containing 0.7% agar before transfer to media where KNO3 and ammonium tartrate from BCDAT medium was replaced with potassium sodium tartrate tetrahydrate. Experimental conditions for nitrogen starvation gene expression analysis and visualization of autophagic particles were performed under continuous light to discriminate the effects of carbon and nitrogen deficiencies. For LY294002 (Sigma-Aldrich, stock solution diluted in DMSO) experiments, 40 μM of the drug was added to solid growth media before it solidified. Protonemata were transferred to LY containing media at the time of darkness treatment initiation, that is, 4 h or 8 h before observation under the microscope (control cells were incubated for 4 h). For Concanamycin A (Santa Cruz Biotechnology sc-202111, stock solution diluted in DMSO) experiments in nitrogen starvation treatment, 0,5 μM of the drug were added to solid growth media before it solidified. Tissue was transferred to this condition 16 h previous observation under the microscope (for the 8 h deficiency time point, tissue was only incubated in Concanamycin A for 8 h). Cells were observed using a Nikon Eclipse Ti confocal microscope (see section Live-cell microscopy of GFP-ATG8-Labeled Autophagic vesicles and image analysis).

### 4.6 Chlorophyll determination and Chlorophyll fluorescence measurements

For chlorophyll content determination, chlorophyll was extracted from harvested samples with 80% (v/v) ethanol and quantified spectrophotometrically at 664 nm and 648 nm, followed by calculation using the previously established formula: c_a_ (μg/ml)= 13.36. A_664_ - 5.19.A_648_ and c_b_ (μg/ml)= 27.43 A_648_ - 8.12.A_648_. The parameters Quantum efficiency of photosystem II photochemistry (ϕPSII = (Fm’- Ft)/Fm’) and maximum quantum yield (Fv/Fm = (Fm -Fo)/Fm) were measured using a pulse amplitude modulated (PAM) fluorometer (FMS2, Hansatech Instruments, Pentney King’s Lynn, UK). Before the measurements, moss colonies were dark adapted for 30 min at room temperature.

### 4.7 Colony density analysis

Solidity index, which measures the density of an object, was constructed as a area/convex hull area ratio using 3 week-old colonies. For area measurements, masks of the colonies were created using FIJI-IMAGEJ software (https://imagej.net/software/fiji/). The convex hull area is the area of the smallest convex shape that contains the colony. Values approaching 1 represent a more solid colony, with a high density; lower values represent colonies with irregular boundaries or containing holes. For quantification of branches per millimeter of main filament, protonemata filaments of 1 and 2 week-old colonies were imaged using an Olympus SZX16 stereomicroscope with a color camera (Olympus DP71) and measured with FIJI-IMAGEJ.

### 4.8 Cell length and growth rate analysis

Cell length measurements were performed on the three-first subapical cells of 7-day-old protonema grown in BCD media, stained with 10 mg. ml^-1^ of Caclcofluor, (Fluorescence brightener 28, Sigma-Aldrich). Calcofluor fluorescence was imaged with a UV filter set. For cell growth rate measurements, protonemal tissue was grown on Petri dishes (30-mm diameter) on BCD media for 7-days. Images of apical chloronemal or caulonemal cells were acquired every 10-15 min during a 2-3-h period. Protonemata cell images were taken with an Olympus BX-61 microscope equipped with a color camera (Olympus DP71). The measurements of cell length and growth rate were made using FIJI-IMAGEJ software.

### 4.9 Cell death measurements

Cell death measurement was performed according to (Castro et al., 2016). Briefly, moss colonies were incubated with 0.05% Evans Blue and after 2 hours tissues were washed 4 times with deionized water to remove excess and unbound dye. Dye bound to dead cells was solubilized in 50% methanol with 1% SDS for 45 min at 50°C and the absorbance was measured at 600 nm. Each biological sample consisted of 3 colonies incubated in 2 mL of the mixture methanol/SDS. Three samples were analyzed per experiment and expressed as OD/g dry weight. Dry weight was measured after drying plant colonies for 18 hours at 65°C.

### 4.10 Gene expression analysis by real-time qRT–PCR

Extraction of plant total RNA was carried out using Quick-Zol Reagent (Kalium Technologies) following the manufacturer’s instructions from 7-day-old ground protonemata grown under the conditions mentioned above. 2 μg of total RNA was treated with 2 units of DNase I (Thermo Fisher Scientific) and then used as a template for cDNA synthesis with RevertAid Transcriptase (Thermo Fisher Scientific) and oligo-dT as primer following the manufacturer’s instructions. qRT-PCR was performed in thermocycler iQ5 Real-Time PCR detection system (Bio-Rad Life Science) using IQ SYBR Green SuperMix (Bio-Rad Life Science), according to the manufacturer’s instruction. The sequences of primers for qRT-PCR are described in **Table S3**. The genes encoding Actin and Ade-PRT were used as controls for the normalization of mRNA content between samples, according to previously published data (Le Bail et al., 2013). Melting curves were obtained to ensure that only a single product was amplified. The relative quantification of the target gene was obtained using Pfaffl method (Pfaffl, 2001).

### 4.11 GFP-ATG8 cleavage assay

For the GFP-ATG8a cleavage assay, 7-day old moss protonemata proteins with the indicated treatments were harvested at the indicated time points were extracted in 100 mM Tris-HCl, 1 mM EDTA, 2% SDS, 100 mM NaCl, 1 mM PMSF and 1 μM E-64D in a relation 4:1 (buffer: fresh weight tissue). Protein concentration was measured by the Lowry method (Lowry et al., 1951) using a SYNERGY H1 microplate reader (Biotek). Equal amounts of total proteins (35 μg) were subjected to 12% SDS–PAGE and to ensure equal loading of protein samples, blots were stained with 0.5% Ponceau Red. Membranes were blocked in Tris-buffered saline (TBS; 20 mM Tris-HCl, 150 mM NaCl, pH7.4) containing 3% (weight in volume, w/v) skimmed milk powder and 0.2% Tween20 for 1h at room temperature, and then incubated with anti-GFP antibodies (Rat monoclonal, ChromoTek) diluted 1/1500 in TBS containing 0.1% Tween overnight at 4C. Anti-Mouse IgG (whole molecule)−Alkaline Phosphatase antibody (Sigma-Aldrich, A3562) diluted 1/5000 was used as the secondary antibody. The autophagic flux was expressed as the relative ratio of free GFP to GFP-ATG8a fusion quantified by ImageJ software in the same sample.

### 4.12 Live-cell microscopy of GFP-ATG8-Labeled Autophagic vesicles and image analysis

Protonemata cells were observed using a Nikon Eclipse Ti confocal microscope. GFP was excited using an Ar488 nm laser and emission was collected at 512/30 nm in the EZ-C1 3.91 software. To include the whole cellular volume, z-stacks of every cell were constructed acquiring 4-5 confocal planes and compiling them with the Max. Intensity method in FIJI-IMAGEJ. Cell area measurements and particles quantification were also performed using FIJI-IMAGEJ.

### 4.13 Quantification of phytohormones by LC-MS/MS

The levels of indole acetic acid (IAA) and salicylic acid (SA) in 7- day old moss protonemta were quantified. The extraction was carried out according to the method by (Otaiza-González et al., 2022), with some modifications. Briefly, 50-100 mg of tissue previously pulverized with liquid N_2_ were weighed, homogenized with 500 μL of 1-propanol/H_2_O/concentrated HCl (2:1:0.002; v/v/v), and stirred for 30 minutes at 4°C. Then, 1 mL of dichloromethane (CH_2_Cl_2_) was applied, stirred for 30 min at 4°C, and centrifuged at 13,000 g for 5 min. The lower organic phase (approx. 1 mL) was collected in vials, which were evaporated in a gaseous N_2_ sequence. Finally, it was re-dissolved with 0.25 mL of 50% methanol (HPLC grade) with 0.1% CH_2_O_2_ and 50% water with 0.1% CH2O2, and stirred slightly with vortex. The LC-MS Waters Xevo TQs Micro (Waters, Milford, MA, USA) was equipped with a quaternary pump (Acquity UPLC H-Class, Waters), autosampler (Acquity UPLC H-Class, Waters), and a reversed-phase column (C18 Waters BEH 1.7 µm, 2.1 x 50 mm, Waters). It was used as a mobile solvent system composed of water with 0.1% CH_2_O_2_ (A) and MeOH with 0.1% CH_2_O_2_ (B), with a correction flow of 0.25 mL/min. The initial gradient of B was maintained at 40% for 0.5 min, and then linearly increased to 100% at 3 min. For identification and quantification purposes, a mass spectrometer Xevo TQ-S micro from Waters (Milford, MA, USA) coupled to the above-mentioned UPLC (LC-MS/MS) was used. The ionization source was used with electrospray (ESI), and the MassLynx Software (version 4.1) was used for data acquisition and processing. The mass spectra of the data are recorded in positive mode. The mass/charge ratio (m/z) for each metabolite were: SA: 137.0 > 65.0 and 137.0 > 93.0; IAA: 176.0 > 103.0 and 176.0 > 130.0; ABA: 263.0 > 153.0 and 263.0 > 219.0. The quantification of the activity was conducted following the calibration curves with the linear adjustment, obtaining the results in a nanogram phytohormone/milligram of fresh weight.

## Data availability statement

The original contributions presented in the study are included in the article/**Supplementary Material**. Further inquiries can be directed to the corresponding author.

## Author contributions

RL and LS conceived the project. RL, LS, and GP designed the experiments. SO-G, PV, and MT performed the hormonal quantification and P.A-R performed the *PpATG8s* promoter network analysis. All remaining experimental work was performed by LS, GP, JF, MP, and FL. LS, GP, JF, FL, GR, AE, CG and RL analyzed the data. LS, GP and RL wrote the manuscript draft and incorporated feedback from all authors. All authors contributed to the article and approved the submitted version.
