# Supplementary material for: Autophagy modulates growth and development in the moss Physcomitrium patens
Source: Front Plant Sci. 2022 Dec 19;13:1052358. doi: 10.3389/fpls.2022.1052358 (PMC9807217; doi:10.3389/fpls.2022.1052358)
Supplement: Supplementary file 1 [file DataSheet_1.docx]

***Supplementary Material***

## Supplementary Figures


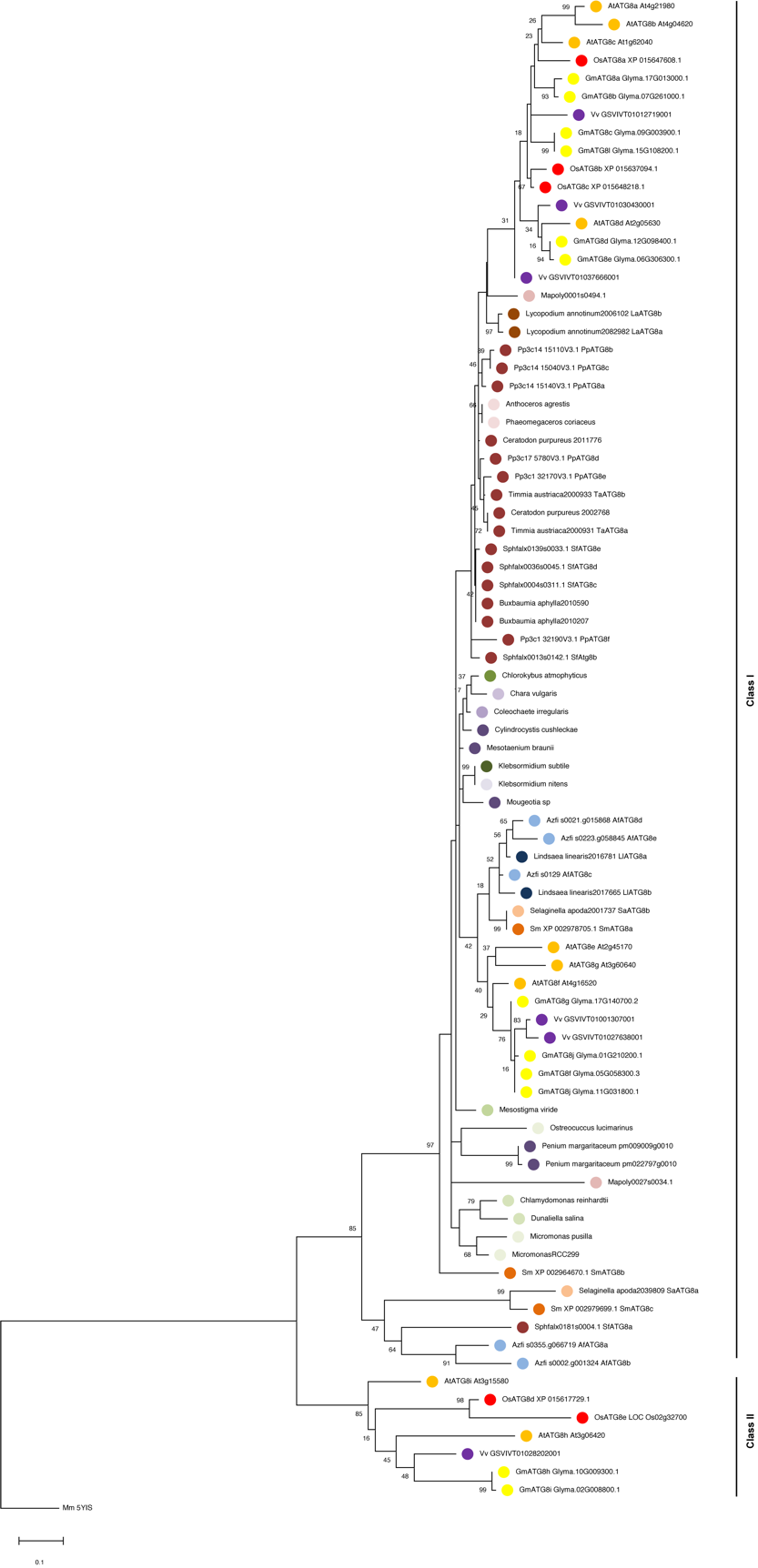


**Supplementary Figure 1.** Phylogenetic tree of ATG8s. The evolutionary history was inferred by using the Maximum Likelihood method and JTT matrix-based model. The percentage of trees in which the associated taxa clustered together is shown next to the branches. The tree is drawn to scale, with branch lengths measured in the number of substitutions per site. This analysis involved 84 amino acid sequences. All positions with less than 95% site coverage were eliminated, i.e., fewer than 5% alignment gaps, missing data, and ambiguous bases were allowed at any position (partial deletion option). Evolutionary analyses were conducted in MEGA X (1).

##
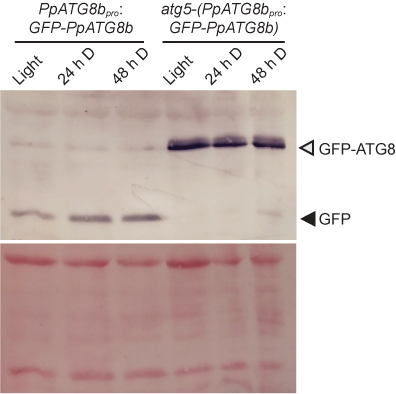
Supplementary Figure 2. The autophagic flux is blocked in *P. patens* *atg5*- (*PpATG8e::GFP-PpATG8e)* loss-of-function mutants. Upper panel: Anti-GFP immunoblot performed using protein extracts of 7-day old protonemata of *PpATG8e::GFP-PpATG8e* or *atg5-PpATG8e::GFP-PpATG8e* line under optimal growth conditions (16 h light, L), or after 24 h or 48 h treatment of darkness. Lower panel: Protein loading control stained with Red Ponceau.


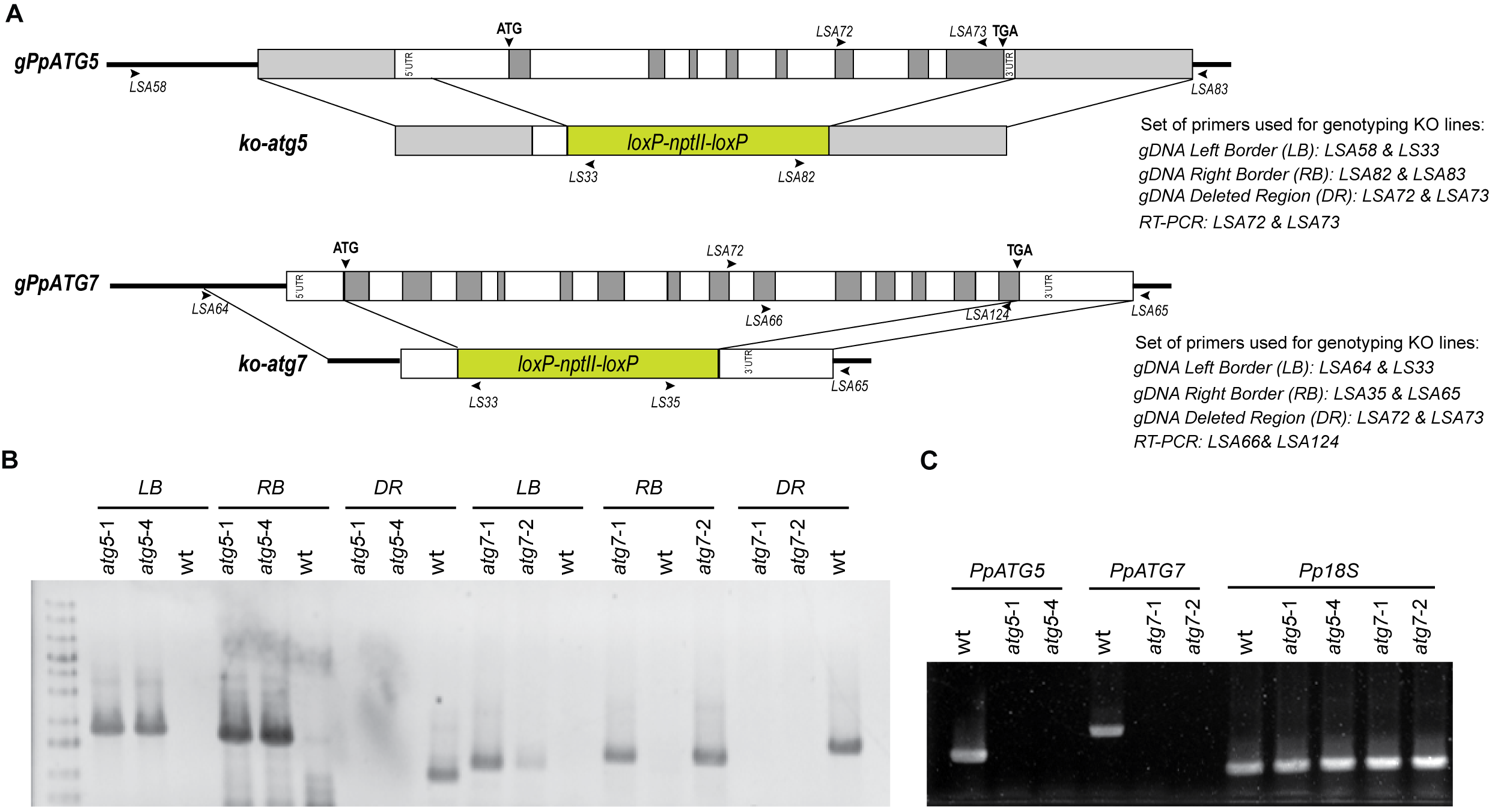


## Supplementary Figure 3. Constructs used for homologous recombination and genotyping *atg* KO plants. (A) Schematic representation of the genomic locus for *PpATG5* and *PpATG7*, and the derived constructs used in this study for the disruption of the gene by homologous recombination. White and grey boxes correspond to exons and introns, respectively. The locations of the primers used for genotyping are shown by arrowheads. Primers sequences are listed in Supplemental Table 2. (B) PCR genotyping analysis of wild-type, *atg5* and *atg7* knockout lines. The location of the primers used for genotyping is shown in A. Gene targeting events were detected by simultaneous PCR amplifications utilizing gene-specific primers external to the targeting construct in combination with outward-pointing primers specific to the selectable marker cassette. PCR genotyping with primers towards the deleted region, amplified only when the *atg5 or atg7* locus is unaltered, but not in the knockout lines. Left border (LB); right border (RB), deleted region (DR). (C) RT-PCR of wild-type, *atg5,* and *atg7* knockout lines showing the absence of *atg* transcripts in the knockout lines.

##
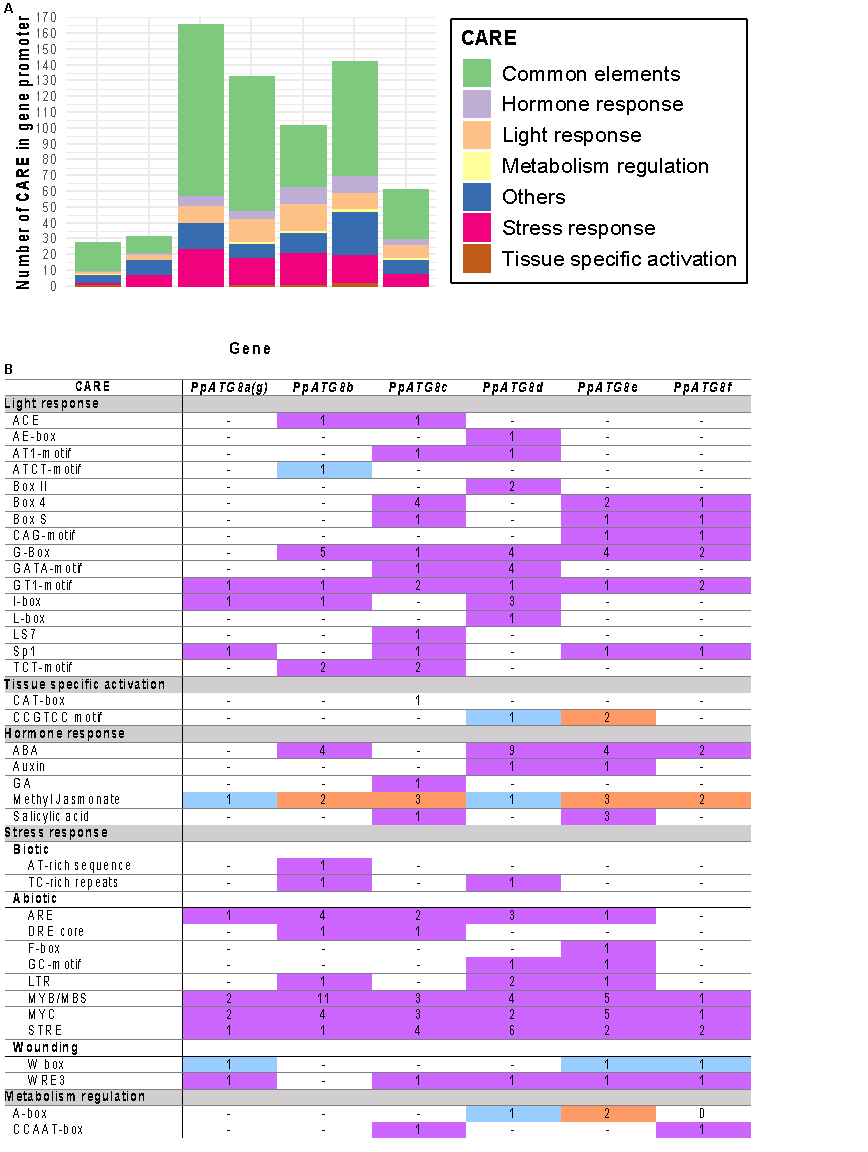


## Supplementary Figure 4. Analysis of Cis Acting Regulatory Elements (CARE) identified in *PpATG8a-f* promoters. (A) Comparison of CARE composition in each promoter region based on their biological functions. PpADE-PRT was used as a reference gene. "Common elements" category includes TATA-box and other promoter-common motifs involved in transcription regulation (activation/repression). Motifs with unknown functions are listed as "Others". (B) List of the elements included in each CARE category ("Common elements" and "Others" are not taken into account). Numbers indicate how many times the element is present in the sequence. CARE not found in the reference gene are shown in light purple; CARE present in a higher number compared to the reference gene are shown in salmon; CARE present in a lower or same number compared to the reference gene are shown in light blue.


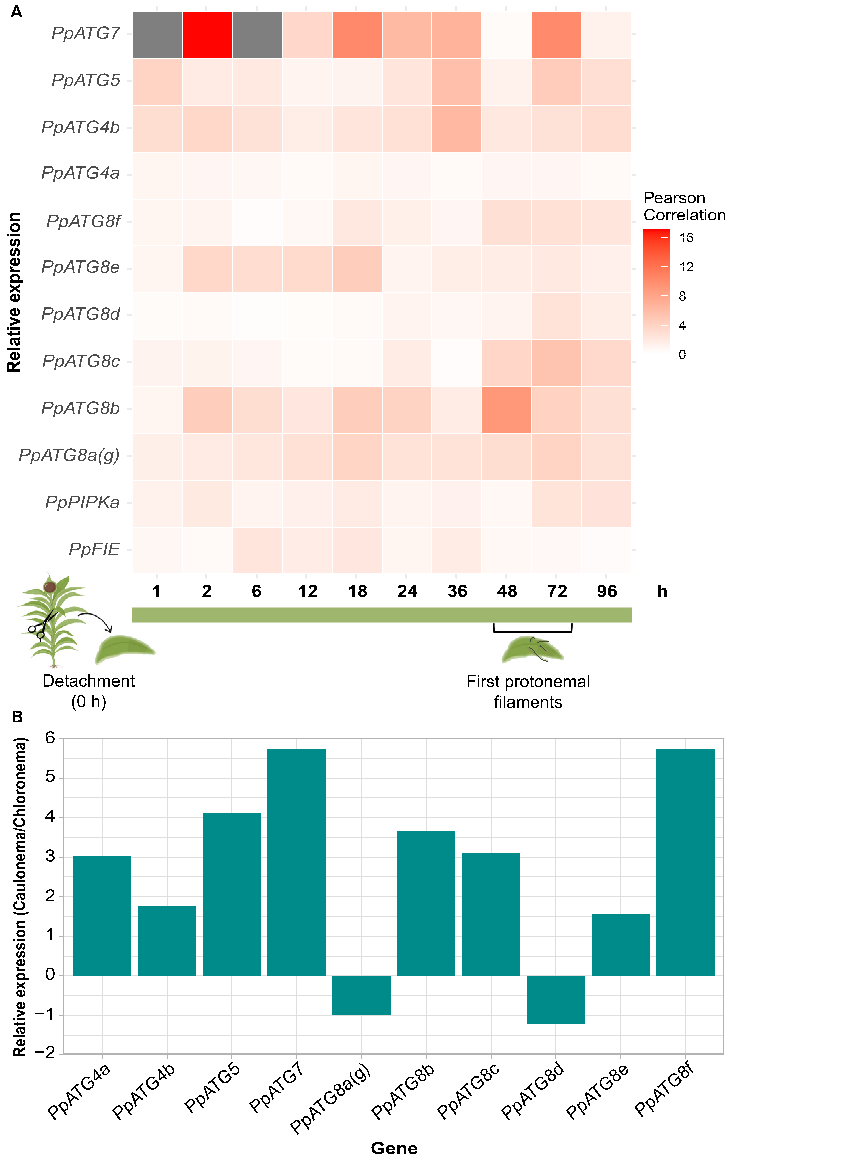


**Supplementary Figure 5**. *In silico* expression analysis of several *PpATG* genes regarding apical growth. (A) Heat map of relative expression during reprogramming and apical growth in *P. patens*. *PpFIE* was used as a reference gene for reprogramming events and *PpPIPKa* as reference for apical growth processes. Values were obtained from PEATmoss database, Combimatrix Leaflet Development gmv1.2 dataset, (2). (B) Relative expression of *PpATG8s* genes indicated as a caulonema to chloronema expression ratio. Data obtained from (3).


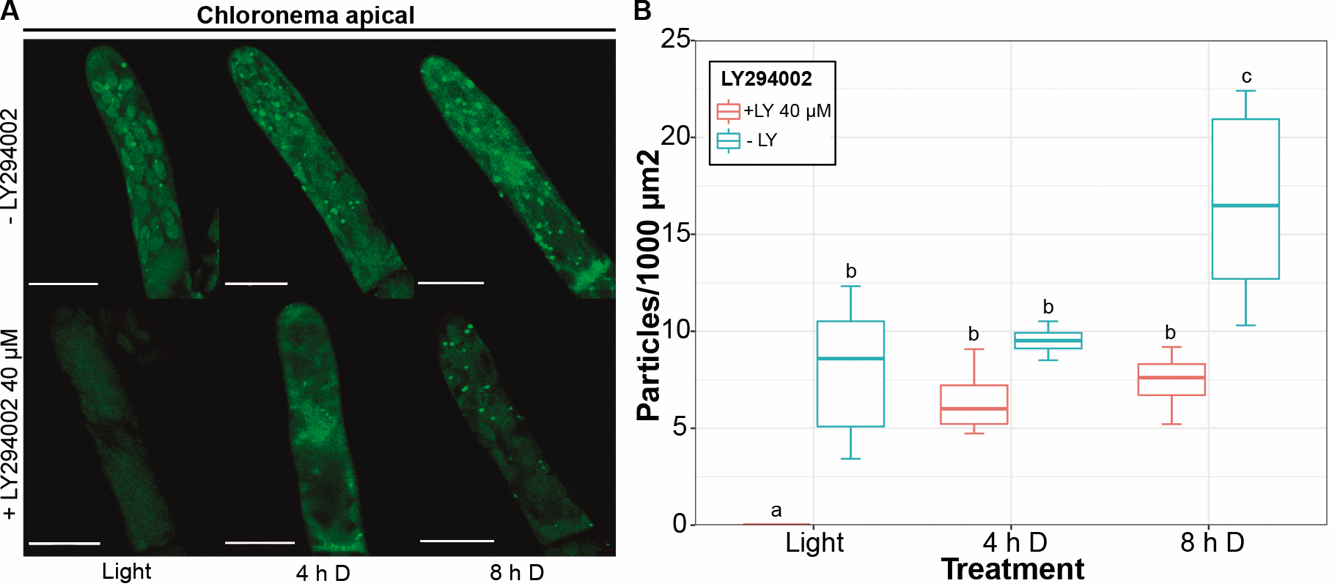


**Supplementary Figure 6.** Quantification of autophagic particles in PpATG8bpro:GFP-PpATG8b chloronema cells treated with LY294002 40 µM. (A) Representative images of chloronema apical cells expressing PpATG8bpro:GFP-PpATG8b under optimal growth conditions (light, 4h, and 8h darkness). Final images were obtained from z-stacks of 4-5 confocal planes. Scale bars: 20 μm. (B) Quantification of autophagic particles. The number of particles was quantified per single cell area and then referred to 1000 μm2. Values represent the mean ± s.d. of the biological replicates. Letters indicate groups with significantly different means (n= 7-10; One-way ANOVA and Tukey’s HSD post hoc test; P < 0.05).

## 2. Supplementary Tables

**Table S1**. List of *ATG* genes from *Physcomitrium patens* and *Arabidopsis thaliana*.

**Table S2**. List of ATG8 proteins across several Viridiplantae species.

**Table S3**. List of primers used in this study.

**Table S4**. Analysis of *PpATG8s* transcription factor binding sites (TFBSs) from the Homer plant database.

**Table S5**. Co-expression network table of *PpATG8s* promoters generated using “NetworkAnalyzer” tool from Cytoscape, keeping nodes with at least two degrees.

1. Kumar S, Stecher G, Li M, Knyaz C, Tamura K. MEGA X: Molecular Evolutionary Genetics Analysis across Computing Platforms. Mol Biol Evol. 2018;35(6):1547-9.

2. Fernandez-Pozo N, Haas FB, Meyberg R, Ullrich KK, Hiss M, Perroud PF, et al. PEATmoss (Physcomitrella Expression Atlas Tool): a unified gene expression atlas for the model plant Physcomitrella patens. Plant J. 2020;102(1):165-77.

3. Xiao L, Wang H, Wan P, Kuang T, He Y. Genome-wide transcriptome analysis of gametophyte development in Physcomitrella patens. BMC Plant Biol. 2011;11:177.
